# IL-28B is a Key Regulator of B- and T-Cell Vaccine Responses against Influenza

**DOI:** 10.1371/journal.ppat.1004556

**Published:** 2014-12-11

**Authors:** Adrian Egli, Deanna M. Santer, Daire O'Shea, Khaled Barakat, Mohammedyaseen Syedbasha, Madeleine Vollmer, Aliyah Baluch, Rakesh Bhat, Jody Groenendyk, Michael A. Joyce, Luiz F. Lisboa, Brad S. Thomas, Manuel Battegay, Nina Khanna, Thomas Mueller, D. Lorne J. Tyrrell, Michael Houghton, Atul Humar, Deepali Kumar

**Affiliations:** 1 Infection Biology, Department of Biomedicine, University of Basel, Basel, Switzerland; 2 Li Ka Shing Institute of Virology, University of Alberta, Edmonton, Alberta, Canada; 3 Division of Infectious Diseases, University of Alberta, Edmonton, Alberta, Canada; 4 Faculty of Pharmacy, University of Alberta, Canada; 5 Division of Infectious Diseases, Moffitt Cancer Center, Tampa, Florida, United States of America; 6 Department of Biochemistry, School of Translational Medicine, Faculty of Medicine & Dentistry, University of Alberta, Edmonton, Alberta, Canada; 7 Division of Infectious Diseases and Hospital Epidemiology, University Hospital of Basel, Switzerland; 8 Division of Nephrology, University Hospital of Zurich, Zurich, Switzerland; 9 Department of Medicine and Multi-Organ Transplant Program, University Health Network, Toronto, Ontario, Canada; University of Washington, United States of America

## Abstract

Influenza is a major cause of morbidity and mortality in immunosuppressed persons, and vaccination often confers insufficient protection. IL-28B, a member of the interferon (IFN)-λ family, has variable expression due to single nucleotide polymorphisms (SNPs). While type-I IFNs are well known to modulate adaptive immunity, the impact of IL-28B on B- and T-cell vaccine responses is unclear. Here we demonstrate that the presence of the IL-28B TG/GG genotype (rs8099917, minor-allele) was associated with increased seroconversion following influenza vaccination (OR 1.99 p = 0.038). Also, influenza A (H1N1)-stimulated T- and B-cells from minor-allele carriers showed increased IL-4 production (4-fold) and HLA-DR expression, respectively. *In vitro*, recombinant IL-28B increased Th1-cytokines (e.g. IFN-γ), and suppressed Th2-cytokines (e.g. IL-4, IL-5, and IL-13), H1N1-stimulated B-cell proliferation (reduced 70%), and IgG-production (reduced>70%). Since IL-28B inhibited B-cell responses, we designed antagonistic peptides to block the IL-28 receptor α-subunit (IL28RA). *In vitro*, these peptides significantly suppressed binding of IFN-λs to IL28RA, increased H1N1-stimulated B-cell activation and IgG-production in samples from healthy volunteers (2-fold) and from transplant patients previously unresponsive to vaccination (1.4-fold). Together, these findings identify IL-28B as a key regulator of the Th1/Th2 balance during influenza vaccination. Blockade of IL28RA offers a novel strategy to augment vaccine responses.

## Author Summary

Infection with influenza viruses is associated with high morbidity and mortality. Therefore, vaccination is recommended in immunosuppressed patients, however often the post-vaccine induced protection is insufficient. Factors associated with reduced vaccine responses may guide preventive strategies and could offer novel targets for adjuvants. Here, we explore the impact of IL-28B on B- and T-cell responses during vaccination. We found that a single nucleotide polymorphism (minor allele genotype) in the IL-28B gene was associated with a significant increase in the antibody seroconversion rate following influenza vaccination. Interestingly, this SNP reduces the expression of IL-28B. In addition, *in vitro* stimulation of peripheral blood mononuclear cells from patients with the SNPs had increased IL-4 production in CD4 T-cells. As a potential mechanism, we show that recombinant IL-28B inhibits influenza stimulated Th2 cytokine release, B-cell activation/proliferation and H1N1-induced IgG secretion. Next, we developed antagonistic peptides to block the IFN-λ receptor. Pre-treatment with the antagonistic peptides increased *in vitro* B-cell activation and antibody production in healthy individuals and transplant recipients. Together, these findings identify IL-28B as a key regulator of Th1/Th2 balance during influenza vaccination. Blockade of the IFN-λ receptor with antagonistic peptides may offer a novel strategy to augment vaccine responses.

## Introduction

Generation of a protective and durable immune response is the major challenge of effective vaccinations against influenza. On a global scale, infection with influenza viruses is associated with increased morbidity and mortality in elderly persons, pregnant women and immunosuppressed individuals [1]. The primary means to limit this disease is through annual influenza vaccination as recommended [2]. However, annual influenza vaccines are poorly effective in the elderly, and immunocompromised populations [3]–[5]. For example, after organ transplantation, post-vaccine seroconversion rates only approach 30 to 50% [4], [6], [7]. Although this may be a function of diminished adaptive immune responses, there are increasing data that interferons (IFN) may modulate vaccine responses [8]–[12]. Understanding the factors involved in a successful vaccine response and seroconversion will allow optimization of vaccine strategies [13].

The IFN-λ family (Interleukin-28A, -28B, -29, and IFN-λ4) is a recently described class of IFNs with antiviral properties similar to IFN-α and -β [14]–[17]. IFN-λ is known to induce phosphorylation of STAT-1 and -2 via binding to its receptor, which is a heterodimer consisting of the IL-28 receptor alpha subunit (IL28RA) and IL-10 receptor beta subunit (IL10RB) [16]. In addition to their anti-viral effects, one of the IFN-λ family members (IL-29) has been shown to increase Th1 and suppress Th2 cytokine producing T-cells [18]–[21]. Furthermore, IFN-λs induced the development of T-regulatory cells *in vitro* [22], [23]. These findings indicate the substantial role of IFN-λs in immune responses, however, this has not been explored in the context of vaccine responses or influenza infection.

Single nucleotide polymorphisms (SNPs) in IL-28B are divided according to their frequencies in a population. At rs8099917, TT is the major-allele and TG or GG are minor-allele genotypes; at rs12979860, CC is the major-allele and CT or TT are minor-allele genotypes [24]–[27]. We selected these two SNPs as they are commonly described in the literature to affect IL-28B functions.

Since IFN expression is involved in multiple aspects of the immune response, we hypothesized that the effectiveness of vaccination may be modulated by variation in IL-28B expression as a consequence of SNPs. We further explored the possibility that altered expression of IL-28B might be associated with changes in B- and T-cell responses. In this study, we chose to use clinical samples obtained from organ transplant patients. These patients receive lifelong immunosuppression and have impaired adaptive immune responses to vaccination. Therefore, any impairment of the innate immune response which alters stimulation of adaptive immunity, is likely to take on greater importance. This population also stands to have the greatest gain from strategies to augment vaccine responses.

Here we show that transplant patients that carry minor-alleles in the IL-28B (rs8099917, TG or GG) gene have significantly higher rates of seroconversion following influenza vaccination. PBMCs from transplant patients with the minor-allele expressed less Th1 cytokines, had more IL-4 producing H1N1-specific T-cells, and higher HLA-DR activation marker expression on naive B-cells than those from major-allele carriers. Consistent with these findings, *in vitro* addition of IL-28B to PBMCs increased Th1 cytokine expression, decreased Th2 cytokines, and decreased H1N1 stimulated B-cell proliferation and IgG production. We also show healthy volunteer PBMCs from minor-allele carriers stimulated with H1N1 expressed less IL-28B. Antagonistic peptides designed to block the interaction between IL-28B and its receptor, reversed these effects and could potentially be used as a novel class of vaccine adjuvants.

## Results

### SNPs in IL-28B affect vaccine responses in organ transplant recipients

#### Transplant recipients carrying the minor-allele of IL-28B have increased seroconversion following influenza vaccination

We obtained blood samples from a cohort of transplant recipients (n = 196) on maintenance immunosuppression that were originally recruited as part of a randomized controlled trial of intradermal versus intramuscular influenza vaccination [28]. Patient demographics are shown in S1 Table. Patients were genotyped for IL-28B SNPs (rs8099917 and rs12979860) and the genotypic distribution is shown in S2 Table and was in equilibrium with general population distribution [24]–[27].

**Figure 1:**
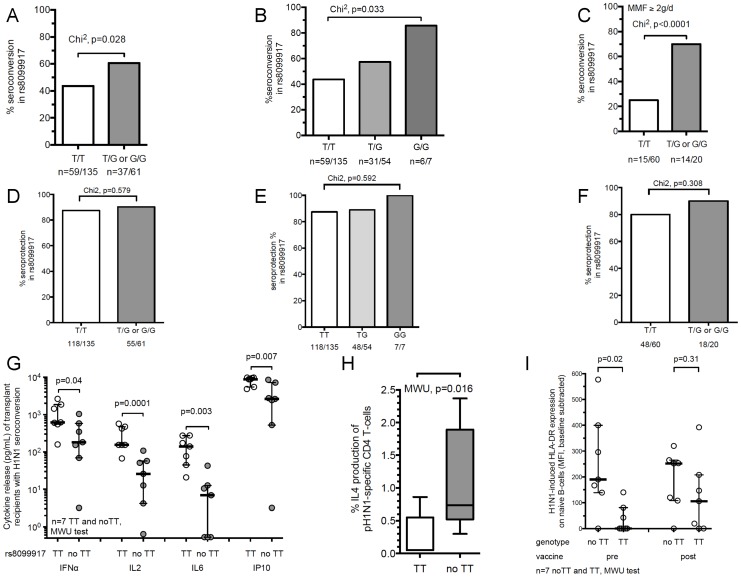
IL-28B genotype significantly impacts Influenza H1N1-stimulated immune responses in transplant recipients. (A) Percent seroconversion to at least one influenza strain antigen in T/T (major) versus T/G or G/G (minor) IL-28B SNP in transplant recipients (rs8099917). Chi^2^ test was used to determine significance. (B) Percent seroconversion to at least one influenza strain antigen in T/T vs. T/G vs. G/G IL-28B SNP in transplant recipients (rs8099917). (C) Percent seroconversion to at least one influenza strain antigen in T/T (major) versus T/G or G/G (minor) IL-28B SNP in transplant recipients (rs8099917) receiving 2 g or more mycophenolate mofetil (MMF) per day. (D) Percent seroprotection to at least one influenza strain antigen in T/T (major) versus T/G or G/G (minor) IL-28B SNP in transplant recipients (rs8099917). Chi^2^ test was used to determine significance. (E) Percent seroprotection to at least one influenza strain antigen in T/T vs. T/G vs. G/G IL-28B SNP in transplant recipients (rs8099917). (F) Percent seroprotection to at least one influenza strain antigen in T/T (major) versus T/G or G/G (minor) IL-28B SNP in transplant recipients (rs8099917) receiving 2 g or more mycophenolate mofetil (MMF) per day. (G) Analysis of H1N1-stimulated Th1 cytokine release in transplant recipients with seroconversion against H1N1 Influenza from post–vaccine samples stratified according to IL-28B minor or major-allele genotypes. The rs8099917 TT (major allele, n = 7) compared to non-TT (minor allele, n = 7) genotypes are shown. Peripheral blood mononuclear cells (PBMCs) were stimulated overnight with inactivated Influenza A/H1N1 (0.3 µg/mL hemagglutinin). The expression profile of 17 cytokines was determined using a luminex-based platform. Key representative Th1 cytokines are shown. Mann Whitney U (MWU)-test determined statistically significant differences between groups; bars show median values (C–F). For some individuals the cytokine values were below the limit of detection and thus were given values of the lowest value in the linear range of the assay. (H) Frequency of post-vaccine H1N1-specific Interleukin (IL)-4 producing CD4^+^ T-cells in transplant recipients with seroconversion according to IL-28B genotype (rs8099917, n = 7 each). For flow cytometry gating strategy see S4 Figure. In PBMCs with a TT genotype 28.6% showed a >2-fold increase from unstimulated samples vs. H1N1 stimulated samples. In PBMCs with a no-TT genotype 85.7% showed a >2-fold increase from unstimulated samples vs. H1N1 stimulated samples (p = 0.031). (I) HLA-DR surface expression (mean fluorescent intensity (MFI)) on H1N1-stimulated naïve B-cells (CD20^+^CD27^−^) in transplant recipients stratified according to the IL-28B genotype (rs8099917) for only those who seroconverted. PBMCs were stimulated with inactivated H1N1 influenza overnight. Baseline values represent non-antigen controls.

We found that minor-allele carriers for the SNP rs8099917 (TG or GG) were significantly more likely to undergo seroconversion to at least one antigen of the influenza vaccine (OR 1.99, 95% CI 1.07–3.69; Fig. 1A). This effect was even higher in a homozygous minor-allele genotype, where the GG-genotype resulted in a seroconversion rate of 85.7% (Fig. 1B). S1A–C Figure shows that the HAI geometric mean titers for pH1N1, H3N2 and Influenza B before and after vaccination are higher in TG or GG (minor allele carriers) versus TT (major allele carriers). For pH1N1, the post-vaccine geometric mean titers of patients with the TT genotype were 66.2, for TG 93.8, and for GG 113.2. The median fold changes from pre- to post-vaccine were 2-fold in TT genotypes, 3-fold in TG and 4-fold in GG. For H3N2, the post-vaccine geometric mean titers of patients with the TT genotype were 44.5, for TG 56.6, and for GG 80.0. The median fold changes from pre- to post-vaccine were 2-fold in TT genotypes, 2-fold in TG and 4-fold in GG. For Influenza B, the post-vaccine geometric mean titers of patients with the TT genotype were 31.9, for TG 43.1, and for GG 60.5. The median fold changes pre- to post-vaccine were 1-fold in TT genotypes, 1-fold in TG and 1.5-fold in GG (S1A–C Figure). The immunosuppressive drug dosages were not significantly different between the genotypic groups (S1 Table). This response was primarily driven by seroconversion to influenza A/H1N1 and H3N2 (S3 Table).

The association between genotype and seroconversion was most pronounced in patients receiving comparatively more potent immunosuppressive therapy (i.e., those patients receiving ≥2 g daily dose mycophenolate mofetil (MMF)). In this group, minor-allele carriers for SNP rs8099917 showed markedly higher seroconversion rates (OR 7, 95% CI 2.3–21.5; p<0.0001; n = 80; Fig. 1C).

When analysing seroprotection to at least one antigen, both the baseline and post-vaccination seroprotection rates were high (71.2% for prevaccination and 88.2% post-vaccine seroprotection to at least one antigen). No significant differences were seen between genotypes likely due to the high rate of baseline seroprotection to at least one antigen (Fig. 1D–F). To allow for better interpretation of seroprotection date we analysed seroprotection to at least two vaccine antigens as an outcome. In this analysis, the minor allele group (for rs8099917) had a trend towards greater seroprotection (p = 0.062). In the subgroup of patients receiving MMF≥2 g daily, seroprotection to at least two antigens was significantly greater in the minor allele carriers (p = 0.038) (S1D–F Figure).

No statistically significant difference in vaccine response was evident for the SNP rs12979860 (S4 Table). Therefore, the remaining experiments were performed focusing on the SNP rs8099917.

#### Seroconversion in transplant recipients, the balance of Th1/Th2 cytokine release and B-cell activation is modulated by IL-28B

We next determined whether the IL-28B SNP (rs8099917 TT versus TG/GG) exerted distinct effects on T- and B-cell activation within the transplant patient cohort. In a subset of vaccine recipients (n = 47), we obtained peripheral blood mononuclear cells (PBMCs) both pre- and post-vaccination and stimulated them with the same inactivated influenza A/H1N1 virus as contained in the vaccine. Of these, 16 (34%) seroconverted using the HAI assay after vaccination. In the subgroup that seroconverted, cytokine profiling of H1N1-stimulated PBMCs post-vaccination showed that Th1-cytokines and associated chemokines, such as IFN-α, interleukin (IL)-2, IL-6, and IFN-γ induced protein (IP)-10, were expressed at significantly lower levels in minor-allele genotype patients (rs8099917, TG and GG; Fig. 1G). S5 Table provides a comparison of post-vaccination cytokine release dependent on genotype independent of seroconversion. A heat map and principal component analysis (PCA) including all samples indicated that pre- and post-vaccine samples had significant differences in Th1/Th2 cytokine expression according to the IL-28B genotypes (TT versus TG/GG). For example IL-5 was relatively 2.9-fold higher expressed in minor-allele carriers, whereas IL-2 was relatively 4.3-fold less expressed in minor-allele carriers. The comparison of the cytokine expression profiles related to seroconversion status and genotype demonstrate that minor-allele carriers have a markedly lower Th1- compared to a higher Th2-response. This shift is even further increased in post-vaccine samples (S1G Figure). The differences in expression dynamics of cytokines and clustering according to genotypes are highlighted also in a two-dimensional PCA (S1H–I Figure). In addition, minor-allele transplant patients (TG/GG) with H1N1-seroconversion had significantly higher frequencies of H1N1-stimulated IL-4-producing CD3^+^CD4^+^ T-cells compared to major-allele genotype patients (TT) in post-vaccine samples (p = 0.007, Fig. 1H). These data indicate that minor-allele carriers have significantly lower H1N1-stimulated Th1- but higher Th2 cytokine release compared to major-allele carriers.

We also studied B-cell phenotypes in the context of the rs8099917 IL-28B genotype because a major protective outcome of influenza vaccination is activation of antibody producing B cells. Most strikingly, minor-allele carriers with seroconversion had significantly higher expression of H1N1-induced HLA-DR expression compared to major-allele carriers (Fig. 1I, TG/GG versus TT).

#### Recombinant IL-28B decreases influenza induced early Th2 cytokine production, B cell proliferation and IgG production

**Figure 2:**
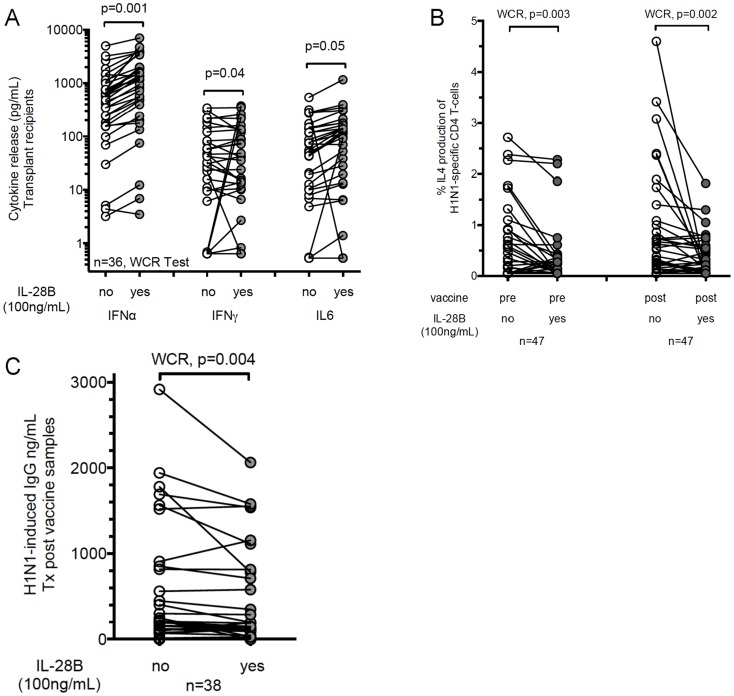
Recombinant IL-28B inhibits Influenza H1N1-induced Th2 response and B cell activation in transplant recipients. (A) Analysis of H1N1-stimulated Th1 cytokine release in transplant recipients (n = 36) from post–vaccine samples in relation to IL-28B pre-treatment. Peripheral blood mononuclear cells (PBMCs) were pre-treated with recombinant IL-28B (100 ng/mL) for two hours prior to overnight stimulation with inactivated Influenza A H1N1 (0.3 µg/mL hemagglutinin). The expression profile of 17 cytokines was determined using a luminex-based platform. Key representative Th1 cytokines are shown. Wilcoxon matched-pairs signed rank (WCR)-test determined statistically significant differences between groups. Before-after plots are shown where each dot is a different patient. (B) PBMCs from transplant recipients were pre-treated with recombinant IL-28B (100 ng/mL) for two hours prior to overnight stimulation. Frequencies of H1N1-specific IL-4-producing CD4^+^ T-cells were measured by intracellular flow cytometry as described in Methods. Data from 47 transplant recipients are shown in PBMC collected pre- and post-vaccine. (C) PBMCs from transplant (Tx) recipients were pre-treated with recombinant IL-28B (100 ng/mL) for two hours prior to 5-day stimulation with H1N1. The production of H1N1-induced IgG is shown according to pre-treatment groups. Data from 38 transplant recipients are shown.

Since previous studies had indicated that minor allele carries expressed lower levels of IL-28B [25], [29]–[33], we hypothesized that adding exogenous recombinant IL-28B to PBMCs should mimic the major-allele phenotype (TT), i.e. increase Th1- and decrease Th2-responses. First, we measured the *in vitro* effect of IL-28B on early cytokine-production by H1N1-stimulated PBMCs from transplant recipients using overnight stimulation assays. In samples pre-treated with IL-28B, we observed a significant increase in H1N1-stimulated Th1-cytokine expression (IFN-γ, IL6; Fig. 2A, S6 Table). In concordance with our previous findings, pre-treatment of transplant recipients' PBMCs with IL-28B prior to H1N1-stimulation led to a >2-fold reduction in IL-4 production by CD3^+^CD4^+^ T-cells (Fig. 2B). Interestingly, the effect was particularly pronounced in patients with a minor allele genotype (TG or GG) (S2 Figure). Further, in transplant recipients, we showed that recombinant IL-28B significantly decreased H1N1-induced IgG production (p = 0.004) (Fig. 2C).

### Healthy volunteer studies

#### SNP in IL-28B (rs8099917) is associated with lower mRNA expression of IL-28B in H1N1 stimulated PBMCs from healthy volunteers

**Figure 3:**
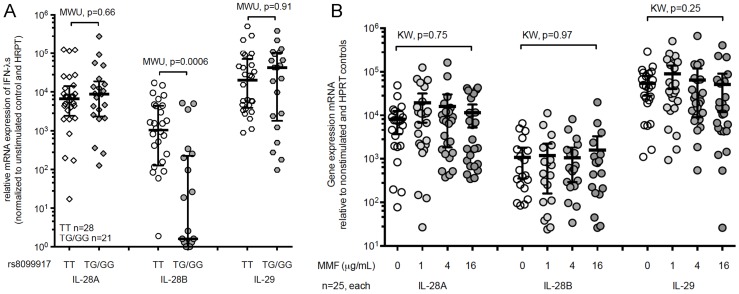
The minor-allele IL-28B genotype is associated with significantly lower expression of IL-28B, but not IL-29 or IL-28A mRNA in healthy volunteers. (A) PBMCs from healthy volunteers were stimulated for six-hours with H1N1. mRNA-expression of IL-28A, IL-28B and IL-29 were normalized to non-stimulated controls and HPRT expression. Major (rs8099917 TT, n = 28) compared to minor (rs8099917 non-TT, n = 21) allele genotype. MWU-test determined statistically significant differences between groups. Bars show median values with interquartile range. (B) PBMCs from healthy volunteers (n = 25) were pre-treated with mycophenolate mofetil for 2 h prior to stimulated for six-hours with H1N1. mRNA-expression of IL-28A, IL-28B and IL-29 and statistical analysis were performed using the Kruskal Wallis (KW) test.

In addition to examination of the effect of IL-28B in our transplant patient cohort, we also wanted to confirm our findings in a non-immunosuppressed cohort and to explore manipulation of the IL-28B system using healthy volunteer cells. We therefore examined the association between the IL-28B genotype (rs8099917) and H1N1-stimulated expression of IL-28B in a cohort of healthy volunteers (HV, n = 28 TT-genotype, n = 21 non-TT genotype). We determined the relative expression of IL-28A, IL-28B, and IL-29 mRNA in PBMCs stimulated with influenza A/H1N1. rs8099917 minor-allele carriers had significantly reduced mRNA-expression of IL-28B whereas IL-28A and IL-29 were not affected (p = 0.0006; Fig. 3A). Plasmacytoid dendritic cells (pDC) have recently been shown to be one of the major producers of IL-28B [34]–[36]. The frequencies of pDCs were comparable between major- and minor-allele groups in the recruited HVs (major-allele carriers 0.39% vs. minor-allele carriers 0.45%; p = 0.713), suggesting that the changes in expression were not due to frequency differences. Second and very important, in a subset of PBMCs from healthy volunteers (n = 25), we pre-treated immune cells with the immunosuppressive drug mycophenolate mofetil (MMF). We found no difference in IFN-λ expression profiles at various concentrations of MMF (Fig. 3B). This suggests that the impact of the genotype is an independent factor associated with influenza-induced IFN-λ gene expression.

#### IL-28B decreases late Th2 cytokines, B cell proliferation, and IgG production in healthy volunteers

**Figure 4:**
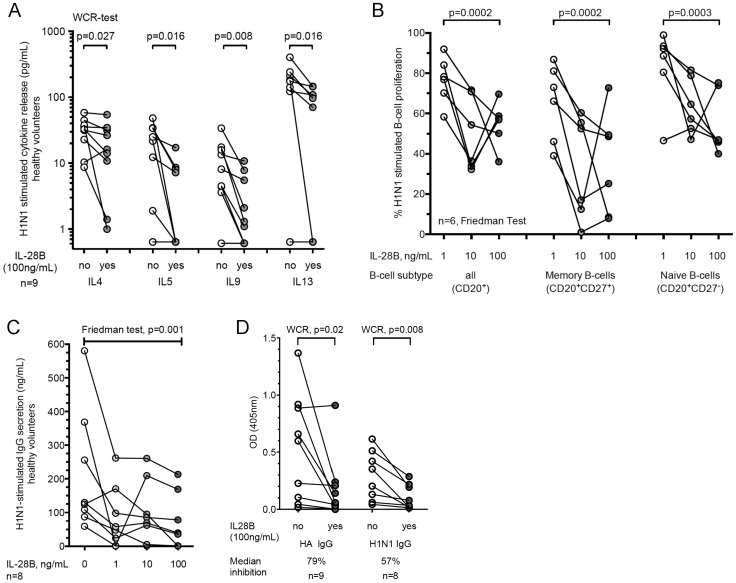
Recombinant IL-28B inhibits Influenza H1N1-induced Th2 response and B cell activation and IgG production in healthy volunteers. (A–D) PBMCs from healthy volunteers were pre-treated with 1 ng/mL, 10 ng/mL, or 100 ng/mL rIL-28B for two hours prior to 5-day (A–C) or 7-day (D) stimulation with H1N1. Kruskal Wallis-test was used to determine statistically significant differences. (A) Cytokine profile after stimulation with H1N1 according to pre-treatment groups (n = 9). (B) B-cell proliferation was quantified using Cell Trace Violet proliferation dye staining. CD20 and CD27 served as cell-type and memory markers, respectively (n = 6). (C) Total amount of IgG in supernatants in an independent cohort of HVs was determined using by ELISA (n = 8). (D) The amount of H1N1- and HA-H1N1-specific IgG in supernatant was determined by ELISA (n = 9, and n = 8 (1 donor did not produce HA-specific IgG) respectively). The reduction of pre-to-post-treated PBMCs was calculated.

Since MMF may influence Th-2 cytokines and B-cell responses, we measured the *in vitro* effect of IL-28B on late cytokine-production by PBMCs from HVs using a 5-day stimulation assay (n = 9). The 5-day stimulatory assay allowed for the secretion of Th2 cytokines that are expressed late during the stimulation. We observed a significant decrease in the production of cytokines trophic for B-cells such as GRO, Fractalkine, and Th2-cytokines (IL-4, IL-5, IL-9, and IL-13) (Fig. 4A and S7 Table). An independently recruited cohort of HVs confirmed the key findings of Th1 and Th1-associated cytokines being significantly up-regulated (IFN-α, IL12p40, TNF- α, and IL-6) at early time-points after stimulation. IFN-γ showed a trend for upregulation. In contrast, Th2 and Th2-associated cytokines were down-regulated at later time-points (S3 Figure).

We then investigated the effects of IL-28B on H1N1-stimulated B-cell proliferation and antibody production. IL-28B pre-treated PBMCs from non-immunosuppressed HVs (n = 6) showed a dose-dependent decrease of H1N1-stimulated B-cell proliferation. In particular memory B-cells exhibited a 70% reduction in proliferation capacity (Fig. 4B). This impairment of proliferation was also reflected in a 70% lower H1N1-stimulated IgG antibody production at the highest dose of IL-28B added (Fig. 4C). We also examined virus-specific antibody production against purified H1N1 hemagglutinin and against the whole virions; these were significantly decreased with a median 57% and 79% reduction at the highest dose of IL-28B added, respectively (Fig. 4D). Taken together, we demonstrate that IL-28B is a potent inhibitor of virus-stimulated B-cell activation and antibody production independent of immunosuppression. Based on live/dead staining, the inhibitory effects were not due to toxicity (median % dead cells IL-28B 0 ng/mL: 19.2%; 1 ng/mL: 19.9%; 10 ng/mL: 23.6%; 100 ng/mL: 22.6%; p = 0.92).

#### Antagonistic peptides to IL28RA promote greater influenza induced antibody production

**Figure 5:**
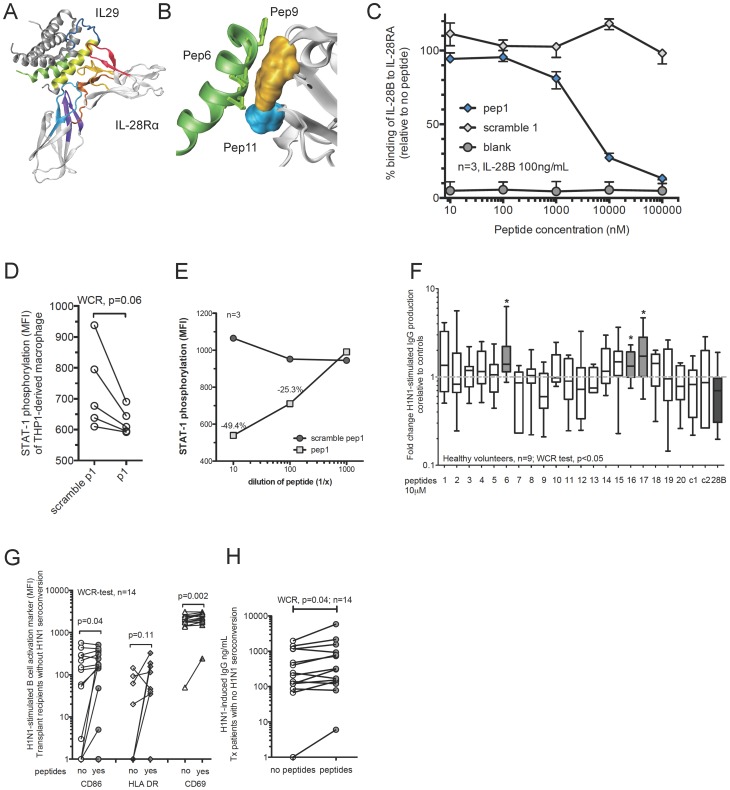
Design of antagonistic peptides against IL-28 receptor alpha subunit (IL28RA) and their effects on H1N1-stimulated B-cell functions. (A) Design of inhibitory peptides based on the crystal structures of IL-29 and the IL28RA. A computer prediction of the interaction between IL-29 and the IL28RA is illustrated. Colored fragments represent proposed sites of significant interaction using a proximity model. (B) Example for detailed *in silico* interaction between peptide 6 and IL-28Rα (peptides 9 and 11). (C) Inhibitory activity of antagonistic peptide 1 against IL-28B binding to IL28RA. ELISA was used to measure the binding of a fixed concentration of IL-28B (100 ng/mL) to IL28RA challenged by increasing concentrations of the inhibitory peptide and control peptide (scrambled version). The data shown is representative of three independently repeated experiments. Whiskers indicate the interquartile range. (D) STAT1 phosphorylation in THP1-derived macrophages treated with peptide 1 and challenged with recombinant IL-28B (100 ng/mL) for 15 min in comparison to scramble peptide control. Data of five individual experiments is shown. Wilcoxon matched-pairs signed rank (WCR)-test was used. (E) STAT1 phosphorylation in THP1-derived macrophages treated with different doses of peptide 1 and challenged with recombinant IL-28B (100 ng/mL) for 15 min in comparison to scramble peptide 1 control. Symbols represent median of three independently repeated experiments. (F) PBMCs from healthy volunteers were pre-treated with peptides for two hours prior to 5-day stimulation with H1N1. In addition recombinant IL-28B (28B) and control peptides (c1, SV40-based peptide; c2, a duck hepatitis B virus based peptide, and various scrambled versions of the peptides) were used. Total IgG in supernatant was determined using an ELISA and fold changes are shown calculated over peptide treatment alone (no H1N1). MWU-test determined statistically significant differences between groups, p<0.05(*). Bars show median and inter-quartile ranges; whiskers indicate 10–90^th^ percentile. (G–H) PBMCs from transplant recipients without successful seroconversion were pre-treated with the IL-28Rα antagonistic peptides (6, 16 and 17) prior to overnight stimulation with H1N1. The mean fluorescence intensity (MFI) of CD86, HLA-DR, and CD69 on B-cells (CD20^+^) was measured using flow cytometry (G). The amount of H1N1-stimulated IgG release was determined by ELISA (H). Wilcoxon matched-pairs signed rank (WCR)-test determined statistically significant differences between groups.

The above findings are highly suggestive of IL-28B possessing a strong immunoregulatory influence on the balance between Th1 and Th2 immune responses, which is in agreement with previous *in vitro* studies for IL-29 [18]–[21], [37], [38]. In turn, this could considerably influence B-cell functions *in vivo*. There are no published crystal structures for IL-28B [39], thus we initially generated a model of the interaction between IL-29 and the IL28-receptor alpha subunit (IL28RA), and then designed peptides based on the known homologies between the different IFN-λs [29]. Peptides with lengths of 14–20 aa were generated to inhibit potential sites of interaction between the ligand and receptor considering close proximity and side-chain interactions (Fig. 5A–B, S5A–B Figure, and S8 Table).

Antagonistic peptides (10 µM) significantly reduced the binding of IL-28B to IL28RA using a competition ELISA assay (S5C Figure). Most of the scramble control peptides did not show a specific antagonistic effect (S5D Figure), however for some unspecific blocking effect could be observed, likely due to the charged nature of the peptide. Of note, the scrambled versions always showed a much lower inhibitory potential compared to the antagonistic peptides.

Antagonistic peptide 1 reduced the binding of IL-28B to IL28RA by 71%, but the scramble version did not block binding at all. Therefore, we explored the blocking capacity of peptide 1 in more detail using an ELISA competition assay with increasing concentrations of antagonistic and scramble peptide control compared to a consistent concentration of IL-28B. We observed a potent specific dose dependent inhibitory blocking effect (Fig. 5C).

The successful interaction between IL28RA, IL10RB and an IFN-λ ligand leads to an immediate phosphorylation of STAT1 and STAT2. Therefore, we used a functional assay based on THP1-derived macrophages to screen the impact of IL28RA blockade on STAT1 phosphorylation. Due to the higher IL28RA expression, THP1-derived macrophages served as an ideal screening model for our designed antagonistic peptides. In our flow cytometry assay, STAT1 phosphorylation peaked in THP1-derived macrophages 15 minutes after IFN-λ and IFN-α stimulation (S5E Figure). We examined the phosphorylation of STAT-1 induced by IL-28B in the presence and absence of the antagonistic peptide “peptide 1”. Treatment with blocking peptide 1 showed a significant reduction in STAT1-phosphorylation upon challenge with IL-28B compared to a scramble peptide control (Fig. 5D). A dose dependent effect of antagonistic peptide 1 on IL-28B-induced STAT phosphorylation could be shown with increasing concentrations (Fig. 5E) in a range similar to that of the ELISA competition assay. This indicates that the peptide not only off-competes the binding to the IL28RA, but also reduces the functional interaction and down-stream signalling of the whole IL28RA/IL10RB/IFN ligand complex.

#### *In vitro* effect of Peptides

We next examined the potential of the peptides to reverse the inhibitory effects of IL-28B signalling on H1N1-stimulated B-cells. We screened all the peptides for their ability to enhance antibody production. PBMCs from HVs pre-treated with an antagonistic cocktail of peptides 6, 16 and 17 showed a significant increase in H1N1-induced IgG production after a five-day stimulation compared to peptide controls (Fig. 5F). Peptides 1, 15 and 18 also showed a strong trend for higher H1N1-induced IgG secretion. Next, we wanted to explore if the effects observed were specifically enhancing H1N1 stimulated IgG production. We tested a broad array of eight different control peptides for their potential to induce changes in the IgG secretion in an expansion protocol. We added fresh peptides twice during the expansion protocol to maximize a potential effect. For various individual IL28RA antagonistic peptides and peptide combinations, the fold change in IgG production was greater than for their respective control peptides. Figure S6 provides results on additional non-functional peptide controls (S6 Figure). This clearly indicates that the increase of H1N1 stimulated IgG production due to the antagonistic peptides is relevant.

Next, we used PBMCs from transplant recipients who had not seroconverted against H1N1-influenza. A combination of peptides 6, 16, and 17 prior to H1N1-stimulation increased the expression of the H1N1-stimulated B-cell activation markers CD86, HLA-DR and CD69 (Fig. 5G) and significantly increased H1N1-stimulated IgG production by 38% compared to peptide controls (median no peptides 226 pg/mL vs peptides 313 pg/mL; Fig. 5H).

## Discussion

We have determined in an immunocompromised transplant population that the presence of the rs8099917 single nucleotide polymorphism (SNP; TG or GG) in the IL-28B gene significantly increases the likelihood of seroconversion to an influenza vaccine especially in those people on high doses of immunosuppression. We also showed that IL-28B affects Th2 and B-cell responses in the context of influenza stimulation. Other important factors associated with B-cell functions such as T-follicular helper cells and IL-21 [40] were not studied.

IL-28B (IFN-λ3) belongs to the family of IFN-lambdas and shares antiviral properties similar to IFN-α via induction of interferon stimulated genes (ISGs) such as MX1 or OAS1 [41]. In addition, IL-28B has been shown to induce IL-12 production in monocytes and macrophages. IL-12 is a key cytokine for the induction of Th1 cells and cytotoxic lymphocytes [42], [43]. SNPs in the IL-28B gene (minor-allele genotypes) have been associated with reduced IL-28B expression [25], [29]–[33], which could impact adaptive immune functions during vaccination. One limitation of our study is that we did not measure serum levels of IL-28B. To the best of our knowledge, no reliable ELISA assay is currently available, which can differentiate between the high sequence homology of IL-28A and IL-28B [44]. In addition, since our study only evaluates patients 30 days post-vaccination, we may not capture the peak of IL-28B secretion. Approximately 40% of Caucasians and 10% of Asian populations carry IL-28B minor-allele genotypes [27]. SNPs in IL-28B have been best studied in the context of response to Hepatitis C therapy. Minor-allele genotypes in IFN-λ signalling have been associated with reduced sustained virologic response of hepatitis C virus (HCV) following IFN-α treatment [24]–[27]. In contrast patients with a IL-28B minor-allele genotype and also at high-risk for primary infection with Cytomegalovirus, had lower frequencies and shorter episodes of primary CMV replication [29], [45]. Patients with minor-allele genotypes of IL-28B showed lower expression of IFN-λ during HCV infection in liver biopsies [25], [30] and during stimulation of PBMCs with CMV [29].

Previous studies have shown that vaccine responses may be influenced by SNPs in interleukin genes. For example, hepatitis B vaccine responses may be influenced by SNPs in the IL-4 gene [46]. SNPs in interleukin genes may also affect humoral and cellular responses to the measles vaccine [47]. In a large cohort of children vaccinated against measles, the rs10853727 SNP in the IL-28B promoter was strongly associated with post-vaccine titers. The major-allele genotype (AA) showed significantly lower measles antibody titers compared to the minor-allele genotypes (AG and GG; median 807 vs. 1004, and 1727 mIU/mL, respectively; p = 0.021) [47].

The effects of SNPs on vaccine responses in the general population may be demonstrated through large-scale genome-wide association studies. However, an immunosuppressed cohort with poor adaptive immunity can be ideal to demonstrate immunogenetic differences in vaccine responses.

We found that the association of the IL-28B SNP with influenza vaccine seroconversion and seroprotection (to at least two vaccine antigens) was even more significant in transplant patients on high doses of mycophenolate mofetil (MMF). MMF is well known to significantly suppress influenza vaccine responses [28], [48] by having an effect on virus-specific Th2 cytokines [49] and on B-cell activation markers, and seroconversion rates [28]. The minor-allele genotype in patients treated with more than two grams MMF per day demonstrated significantly higher seroconversion rates – essentially acting similar to a “rescue” mutation. We speculate that major-allele carrier status with high IL-28B expression in addition to receiving high-dose MMF therapy leads to a “double hit phenomenon” on Th2 responses. As a limitation of our work, we recruited healthy volunteers over multiple months, therefore the numbers within various experiments are variable and some intra-individual variation may be present.

We also determined that the IL-28B rs8099917 SNP affected not only humoral responses to the influenza vaccine but also had a potent effect on cellular responses by modulating the Th1/Th2 cytokine balance. We show that the IL-28B minor-allele genotype is characterized by a predominant Th2-response upon stimulation with H1N1-influenza virus, and is associated with increased B-cell activation (HLA-DR, CD86) and function (IgG production). Although we did not measure H1N1-specific IgG in transplant patients, we do show in healthy volunteers that virus-specific IgG decreases upon pre-treatment with IL-28B *in vitro* at similar inhibition levels. Furthermore, exogenous treatment with IL-28B simulated a major-allele phenotype with significantly reduced Th2 cytokine expression. In PBMCs from healthy volunteers, this phenomenon was independent of MMF treatment. Our findings confirm the previous observation that IL-29 may skew the balance of Th1 and Th2 cytokine towards Th1 and a pronounced cytotoxic T-cell response [18], [19], [21], [38]. Secretion of Th1-cytokines acts as an important suppressor of Th2-cell differentiation [50], [51] via IFN response factors [51] and is associated with lower antibody titers after influenza vaccination [52]. Interestingly, the effect of IL-28B treatment was stronger in minor allele genotypes. The reduced effects in major allele genotypes could be due to a higher baseline expression of IL-28B and saturation of the signalling cascade. This is supported by a study in hepatocytes, where the minor allele genotype was associated with a higher baseline IL28RA expression and increased susceptibility and responses to IFN-λs [53]. A similar mechanism could be present in antigen presenting cells, which in turn has then affects the down-stream effects on T-cells and antibody production.

We further used peptides to inhibit IL-28R signalling. These peptides have previously been described [29]. Inhibition of the IL-28B signalling during vaccination offers the potential to enhance Th2 cytokine release and thereby boost pathogen-specific IgG. It has been previously shown that signalling of the IFN pathway suppresses IgG secretion via increasing Th1 cytokines and a more cytotoxic immune response [52]. In particular antagonistic peptides 1, 6, 16 and 17 are promising candidates with high binding affinities to IL28RA, a strong potential to inhibit binding of IFN-λs and the ability to significantly increase *in vitro* H1N1-induced IgG production. These antagonistic peptides may enable immunomodulation towards Th2 cytokines and have the potential to become a new class of adjuvants by modulating IFN.

An important strength of our study is the use of a clinical cohort including immunosuppressed transplant recipients to confirm our findings in the clinical setting. We then sought to define additional observations to support our clinical findings making our study unique. One limitation of our study is the heterogeneity of the transplant cohort due to a variety of underlying conditions leading to organ failure. However, the immunosuppressive treatment was not significantly different between genotype groups. In addition, we have shown that mycophenolate mofetil (MMF) and the IL-28B major-allele genotype are independent factors for IgG production and that IFN-λ mRNA expression is not influenced by MMF. Another limitation of our study was that at the time of peptide design, only the crystal structure of IL-29 was available and the peptides are therefore based on IL-29 and not IL-28B. However, as IL-29 has a significantly greater binding affinity towards IL28RA compared to IL-28B, this could be advantageous as we are potentially blocking all IFN-λs with greater efficiency. The role of the IL10RB co-recruitment also needs to be further defined.

In summary, SNPs in IL-28B play a key role in vaccine responses especially for influenza vaccine response in immunosuppressed patients. Peptides used to inhibit IFN lambda receptor signalling may play a role in augmenting vaccine responses and as such, represents a novel avenue for developing new adjuvants. Further studies in other populations such as other immunosuppressed populations, elderly persons and healthy individuals would also lead to improved vaccine strategies.

## Materials and Methods

### Patient population

A previously described cohort of immunosuppressed adult solid organ transplant recipients was used for this study [28]. Healthy non-immunosuppressed non-vaccinated volunteers (HV) were recruited as controls. Peripheral blood mononuclear cells (PBMCs) from 47 transplant recipients were available.

### Ethics statement

The study protocols were approved through the University of Alberta research ethics board and written informed consent was obtained from all participants (patients and healthy volunteers).

### Hemagglutination inhibition assay

HAI titers were determined as previously published [28].

### Definitions of vaccine responses

Definitions of vaccine immunogenicity were based on recommendations for annual licensure of influenza vaccine (European Medicines Agency, document: CHMP/VWP/164653/2005). Seroconversion was defined as a ≥4-fold rise in titer from pre-vaccination. Vaccine response was defined as seroconversion to at least one of the three vaccine antigens: influenza A/California/7/2009(H1N1-like), A/Victoria/210/2009(H3N2-like) and B/Brisbane/60/2008 [28].

### Genotyping of polymorphisms in the IL-28B promoter region

SNP genotypes were determined as previously published [29], [54], [55]. Briefly, the probe set to discriminate the rs12979860 discriminates the C and T alleles, where C is the major, and T is the minor-allele [55]. For the rs8099917 SNP, the probe set discriminates the T and G alleles, where T predicts the major, and G is the minor-allele. SNP detection was performed on 6 ng of genomic DNA. S9 Table shows all primer sequences. In each allelic discrimination assay 50 bp synthetic positive control oligonucleotides were included. SNP genotype was determined using the automatic call algorithm in conjunction with the allelic discrimination plot.

### Influenza viruses

For immune stimulation we used formalin inactivated, partially purified A/California/7/2009 (H1N1) (NIBSC, NXMC-X179A, UK). The H1N1 stock contained 50 µg/mL of hemagglutinin protein and was re-constituted in water. For all experiments a final concentration of 0.3 µg/mL was used.

### IL-28A, IL-28B and IL-29 specific TaqMAN gene expression assay

IL-28B primers and probe were designed based on *Homo sapiens* IL-28B mRNA-sequence (NM_172139.2) using Primer3 Input (version 0.4.0) (http://frodo.wi.mit.edu/). The forward primer (IL-28BF1: CAAAGATGCCTTAGAAGAGTCG) spans the exon/exon junction of exons 1 and 2 of IL-28B. The IL-28B-specific probe (IL-28B probe: GCTGAAGGACTGCAAGTGCCG ) is located in the second exon and the reverse primer (IL-28BR1: TCCAGAACCTTCAGCGTCAG) is in the third exon of the IL-28B gene. For IL-28A, the *Homo sapiens* IL-28A mRNA-sequence (NM_172138.1) was utilized. The forward primer (IL-28AF1: CAAAGATGCCTTAGAAGAGTCG) spans the exon/exon junction of exons 2 and 3 of IL-28A. The IL-28A-specific probe (IL-28A probe: GCTGAAGGACTGCAGGTGCCA ) is in exon 3 and the reverse primer (IL-28AR1: TCCAGAACCTTCAGCGTCAG) is found in the fourth exon. Forward and reverse primers were identical for both genes due to high percentage sequence homology. Assay specificity was conferred by a two-nucleotide difference in the probe sequence (underlined). Both assays yield 150 nt products.

Primers and a probe specific for IL-29 were designed based on the *Homo sapiens* IL-29 mRNA-sequence (NM_172140.1). The forward primer (IL-29F1: GGACGCCTTGGAAGAGTCA) spans the exon/exon junction of exons 1 and 2 of IL-29. The IL-29-specific probe (IL-29 probe: CTCAAGCTGAAAAACTGGAGTTGCAGC) is in the second exon of the IL-29 gene and the IL-29 reverse primer (IL-29R1: CCAGGACCTTCAGCGTCA) is in the third exon. The IL-29 assay yields a product of 146 nucleotides. Primers and probes were manufactured by IDT (Integrated DNA technologies, Iowa, USA).

The specificity of the three sets of qRT PCR assays was tested against Invivogen expression plasmids containing complete IL-28A (puno1-hIL-28A), IL-28B (puno1-hIL-28B) and IL-29 (punoIL-29) sequences [16]. The specificity of all qRT PCR assays has been previously assessed [29]. As a house keeping gene HPRT was used [29].

### IgG ELISA for influenza-induced antibodies

Cell-free supernatants were collected from PBMC cultures at indicated time points and stored at −80°C until analysis. An in-house human IgG ELISA assay was developed using antibodies and human IgG standard. In brief, 96 well EIA/RIA plates (Costar) were coated overnight with donkey anti-human IgG antibody at 5 µg/ml. Plates were washed with PBS/0.05% Tween and supernatant samples (diluted 1∶5) or ChromPure Human IgG standard (Jackson Immunoresearch) were added in duplicate for 2 hrs at room temperature. After washing extensively, detection antibody (goat anti-human IgG alkaline phosphatase, 1∶15,000) was added for 1 hr at room temperature. After washing, PNPP substrate was added and the plate was read every 5 min at 405 nm with correction at 570 nm.

Virus-specific IgG production was assessed by coating the previously mentioned plates either with pH1N1 antigen (contained in the vaccine, and used also for T-cell stimulation assays: NIBSC, NXMC-X179A) or purified pH1N1 hemagglutinin (Influenza reagent resource (IRR), FR-559). Supernatants from stimulated PBMC cultures (day 7; diluted 1∶2) were added and the amount of bound antibody was determined as above except supernatants were incubated overnight to increase sensitivity. To confirm specificity, supernatants were added to plates coated with hepatitis B virus surface antigen (Creative Biomart) or HCV E2 antigen (Immunodiagnostics, Inc.) and no signal was detected above background (Median ODs for HA coated wells: 0.607; HBs Ag: 0.00625; HCV E2: 0.00425; and unstimulated sample supernatant with HA coated wells: 0.01175). Results are expressed as absorbance values (405 nm–570 nm) with the plate blank subtracted.

### Design of peptides

Antagonistic peptides were designed as previously published [29]. Briefly, based on previous publications of the crystal structures of IL-29 and the receptor IL28RA (PDB: 3OG4, 3OG6), we determined the amino acid residues, which are in close proximity to mediate interaction between the two molecules. The selected amino acids were compared with the crucial amino acids described in the literature [39], [56]. In order to preserve the interaction domain structure (helix or loop) we selected nearby amino acids that may stabilize the binding domain for inclusion in peptides.

Based on the oligomeric state structure suggested, we designed peptides, which have the potential to bind both IFN-λ and IL28RA. We used the crystal structure of IL-28B oligomer (PDB: 3HHC [56]) focusing on amino acids, whose residues may be involved in the interactions responsible for the formation of the oligomeric state. We then designed peptides to mimic these interaction domains in order to prevent the formation of oligomers. All peptides (and all other reagents) were tested for endotoxin and had <0.25 endotoxin units (EU)/ml.

### ELISA for competition assay

Recombinant IL28RA was coated on an ELISA plate and pre-treated with increasing concentrations of peptides. Next, recombinant, his-tagged IL-28B at a fixed concentration of 100 ng/mL was added. Anti-his secondary antibody was used to determine the relative amount of bound IFN-λ to the IL28RA. These dose-response curves allowed us to determine the effectiveness of binding inhibition of each peptide. Antagonistic peptides were added in a range from 10 nM to 100 µM.

### THP1-derived macrophages

THP1-derived macrophages were generated as previously described [57]. Briefly, THP1 cells were seeded in presence of PMA (100 nM) and incubated at standard conditions in RPMI 10% heat inactivated FCS for 3 days. Then media and non-adherent cells were removed and fresh media without PMA added for another 5 days incubation. These cells (THP1-derived macrophages) where used for the peptide screening assays.

### Flow cytometry

Prior to surface staining, LIVE/DEAD staining was performed (near-IR; Invitrogen). Markers for identifying T-cell subsets were CD3, and CD4. Intracellular cytokine staining was performed according to previously published protocols after overnight stimulation [58]. IL-4 was used as a key representative for Th2 cytokine production. Background (unstimulated samples) were subtracted from stimulated results. All reagents including perm and fixation buffers and antibodies were from eBioscience. Isotype controls have previously been used to establish the assays.

Markers for identification of B-cell subsets were CD20 and CD27, where naïve B-cells are CD20^+^CD27^−^ and memory B-cells are CD20^+^CD27^+^. MHC-II, CD86 and CD69 served as activation markers (Biolegend or eBioscience; see S4 Figure). For B-cell expansion experiments, PBMCs were labeled with Cell Trace Violet proliferation dye (Invitrogen). Labeled PBMCs were washed and resuspended in RPMI with 10% FBS and plated in a 96-well format. Stimulation was according to the respective experimental condition in 5% CO_2_ at 37°C. 2 days after initial stimulation, 50 µL of fresh RPMI was added.

THP1-derived macrophages were stained using STAT1-phosphorylation antibodies (BD Bioscience, AF647 Mouse anti-stat-1 pY701) and respective isotype controls. Macrophages were pretreated with blocking peptides and challenged with IL-28B (100 ng/mL) for 15 min. Then cells were fixed and permeabilized as previously described.

### Cytokine profile

Two luminex-based cytokine profiling kits were used (Eve Technologies, Calgary, Canada). (i) 17-plex: Fractalkine, IFN-α, IFN-γ, GRO, MCP-3, IL-13, sCD40-L, IL-9, IL-1β, IL-2, IL-4, IL-5, IL-6, IP-10, MCP-1, MIP-1α, and TNF-α. (ii) 41-plex: EGF, Eotaxin, FGF-2, FLT3, Fractalkine, G-CSF, GM-CSF, GRO. IFN-α2, IFN-γ, IL-1α, IL-1β, IL-1ra, IL-2, IL-3, IL-4, IL-5, IL-6, IL-7, IL-8, IL-9, IL-10, IL-12p40, IL-12p70, IL-13, IL-15, IL-17, IP-10, MCP-1, MCP-3, MDC, MIP-1α, MIP-1β, PDGF AA, PDGF AB/BB, RANTES, sCD40L, sIL2ra, TGF-α, TNF-α, TNF-β, and VEGF. Our independent experiment for examining a time course of cytokine induction was a custom-plex based on the 17-plex and still run and analyzed by Eve Technologies.

### Cytokine profile analysis

GeneSpring GX version 12 (Agilent Technologies, Canada) was used for cluster and principal component analysis (PCA) of the cytokine data measured in H1N1-stimulated PBMCs. Non-stimulated background samples were subtracted prior. Percentile shift was used as normalization algorithm and baseline transformation was performed to median of all samples. Hierarchical clustering of both conditions and cytokines was done using Euclidean as similarity measure and Centroid as linkage rule. PCA was used to detect major trends in the experimental conditions. 2D PCA Scores are shown for the first and second PCA components. They capture about 90% of the variation and visualize the separation of the conditions. The PCA loading plot indicates the separation in subsets of cytokines (x-axis) and denotes their relative contribution to the principal components on the y-axis. All pre- and post-vaccination samples of 47 transplant recipients were included. The conditions considered for analysis were: pre- vs. post-vaccination and minor- vs. major-allele IL-28B genotype.

### Statistical analysis

Statistical analyses were performed using PASW Statistics (version 20.0, Chicago, Ill.) and GraphPad Prism (version 4.0, La Jolla, CA). Data are shown with median and inter-quartile ranges unless otherwise indicated. Categorical variables were analyzed using a Chi-Square (Chi^2^). Continuous non-normal distributed data (Shapiro Wilk test) were analyzed using a Mann-Whitney U test (MWU) or Kruskal-Wallis test (KW). Paired data were analyzed using Wilcoxon matched pairs rank test (WCR). All tests were two-tailed.

## Supporting Information

S1 Figure **Analysis of H1N1-stimulated antibody and cytokine release in transplant recipients.** (A–C) Pre- to post-vaccine haemagglutination inhibition (HAI) titer according to genotype. The HAI titers are shown for major allele carriers (TT, left) and minor allele carriers (no TT, right) for pH1N1 (A), H3N2 (B), and Influenza B (C). A total of 196 transplant recipients is shown. (D–F) Seroprotection to at least two vaccine antigens. (D) Percent seroprotection to at least two influenza strain antigens in T/T (major) versus T/G or G/G (minor) IL-28B SNP in transplant recipients (rs8099917). Chi^2^ test was used to determine significance. (E) Percent seroprotection to at least two influenza strain antigens in T/T vs. T/G vs. G/G IL-28B SNP in transplant recipients (rs8099917). (F) Percent seroprotection to at least two influenza strain antigens in T/T (major) versus T/G or G/G (minor) IL-28B SNP in transplant recipients (rs8099917) receiving 2 g or more mycophenolate mofetil (MMF) per day. (G–I) Supernatants of H1N1 stimulated PBMCs from transplant recipients (n = 47) was collected after 18 h. A luminex-based cytokine profile analysing 17 cytokines was performed. (G) Clustering analysis comparing IL-28B genotype pre- and post-vaccine (vacc.). (H) Principal component analysis showing PCA scores dependent on IL-28B genotype and pre- vs. post-vaccine state (2-dimensional). (I) Principal component analysis showing PCA loadings of cytokines dependent on IL-28B genotype and pre- vs. post-vaccine state (2-dimensional).
(TIFF)

S2 Figure **Frequency of IL-4-producing CD4 T-cells and impact of pre-treatment with IL-28B.** The differential effect of IL-28B genotype background in PBMCs before and after vaccination is shown. In particular, PBMCs with a minor allele background (no TT) show a strong sensitivity to IL-28B. Wilcoxon matched pairs rank test was used to test for significant difference. Data from 45 transplant recipients is shown.
(TIFF)

S3 Figure **A cytokine profile with pH1N1 stimulation +/− IL-28B pre-treatment (100 ng/ml) was performed.** The time-points were collected within the same experiments. For the first 3 HVs studied, d+5 sample was not collected. Overall, the time-points d+1, d+3, d+7 were available in total 10 HVs and for d+5 in 7 HVs. Wilcoxon matched pairs rank test was used to test for significant difference.
(TIFF)

S4 Figure **Gating strategy for flow cytometry analysis of H1N1-specific T-cell and B-cell responses.** (A) After exclusion of singlets and dead cells, either T-cells (CD3^+^CD4^+^), or B-cells (CD20^+^CD27^−^, naïve B-cells; CD20^+^CD27^+^, memory B-cells; CD20^+^CD27^++^, plasma cells/plasmablasts) were gated. In T-cells, intracellular cytokine staining for IL-4^+^ was determined. Cytokine producing T-cell subsets were expressed as the % frequency of overall CD3^+^CD4^+^ T-cells. In B-cells, surface staining with CD86, HLA-DR and CD69 was determined. Mean fluorescence intensity was used to express the surface expression of the respective activation marker. Background expression (non-stimulated cells) was subtracted for all experiments. (B) Examples of IL-4 producing H1N1-stimulated and non-stimulated CD4+ T-cells are shown. For further analysis the non-stimulated background sample was subtracted.
(TIFF)

S5 Figure **Design of antagonistic peptides to IL-28 receptor (IL28RA) and their binding affinity.** (A) Exampled of detailed *in silico* interaction focusing on peptide 3 and IL28RA. (B) Exampled of detailed *in silico* interaction focusing on peptide 1 and IL28RA. (C) Inhibitory activity of peptides against IL-28B to IL28RA. ELISA was used to measure the binding of a fixed concentration of IL-28B (100 ng/mL) to IL28RA challenged by a fixed concentration of antagonistic peptides (10 µM). Bars indicate median values, whiskers inter-quartile ranges. The graph represents three independently repeated experiments. (D) Inhibitory activity of scrambled peptides against IL-28B to IL28RA. ELISA was used to measure the binding of a fixed concentration of IL-28B (100 ng/mL) to the IL28RA challenged by a fixed concentration of control peptides (10 µM; s, scramble). The median value representing three independently repeated experiments is shown. Whiskers indicate the interquartile range. (E) Time-course of STAT-1 phosphorylation in THP1-derived macrophages peak at 15 min. STAT-phosphorylation is expressed in mean fluorescent intensities of 5 independent experiments, mean and SEM is shown.
(TIFF)

S6 Figure **Effects of control peptides on H1N1 stimulated IgG production.** Various control peptides were added twice to H1N1 stimulated PBMCs to maximize a potential unspecific stimulatory effect during the expansion phase. No unspecific effect could be observed. Control peptides are abbreviated as “ctrl”. Ctrl 1–3, “DV2”, “DV8”, “DV10” are peptides based on the NS5A of HCV, Ctrl 4, “DHBV” is a peptide based on the pre S region of duck hepatitis B virus encoded proteins. Ctrl 5, “Pen” is penetratin from drosophila, and Ctrl 6, “SV40” is based on the large T protein in simian virus 40. All control peptides are unrelated to IL-28A, IL-28B, IL-29 or IL28RA or IL10RB.
(TIFF)

S1 Table **Demographics of transplant cohort.**
(DOCX)

S2 Table **Distribution of allele frequencies of IL-28B genotypes (rs8099917 and rs12979860) in transplant cohort.**
(DOCX)

S3 Table **Influenza strain specific seroconversion rates in relation to IL-28B genotype.**
(DOCX)

S4 Table **Seroconversion rates to at least one antigen of influenza vaccine in relation to IL-28B genotype.**
(DOCX)

S5 Table **The effects of IL-28B genotype on production of cytokines by transplant recipient PBMCs in response to** ***in vitro*** **overnight influenza H1N1 stimulation.**
(DOCX)

S6 Table **The effects of IL-28B treatment on production of cytokines by transplant recipient PBMCs in response to** ***in vitro*** **overnight influenza H1N1 stimulation.**
(DOCX)

S7 Table **The effects of IL-28B addition on production of cytokines by healthy volunteer PBMCs in response to** ***in vitro*** **influenza H1N1 stimulation.**
(DOCX)

S8 Table **Amino acid sequences of Interferon-λ ligand or receptor-based inhibitory peptides.**
(DOCX)

S9 Table **Primer and probe sequences for IL-28B SNP genotyping.**
(DOCX)
