# Supplementary figures and images for: IL-28B is a Key Regulator of B- and T-Cell Vaccine Responses against Influenza
Source: PLoS Pathog. 2014 Dec 11;10(12):e1004556. doi: 10.1371/journal.ppat.1004556 (PMC4263767; doi:10.1371/journal.ppat.1004556)

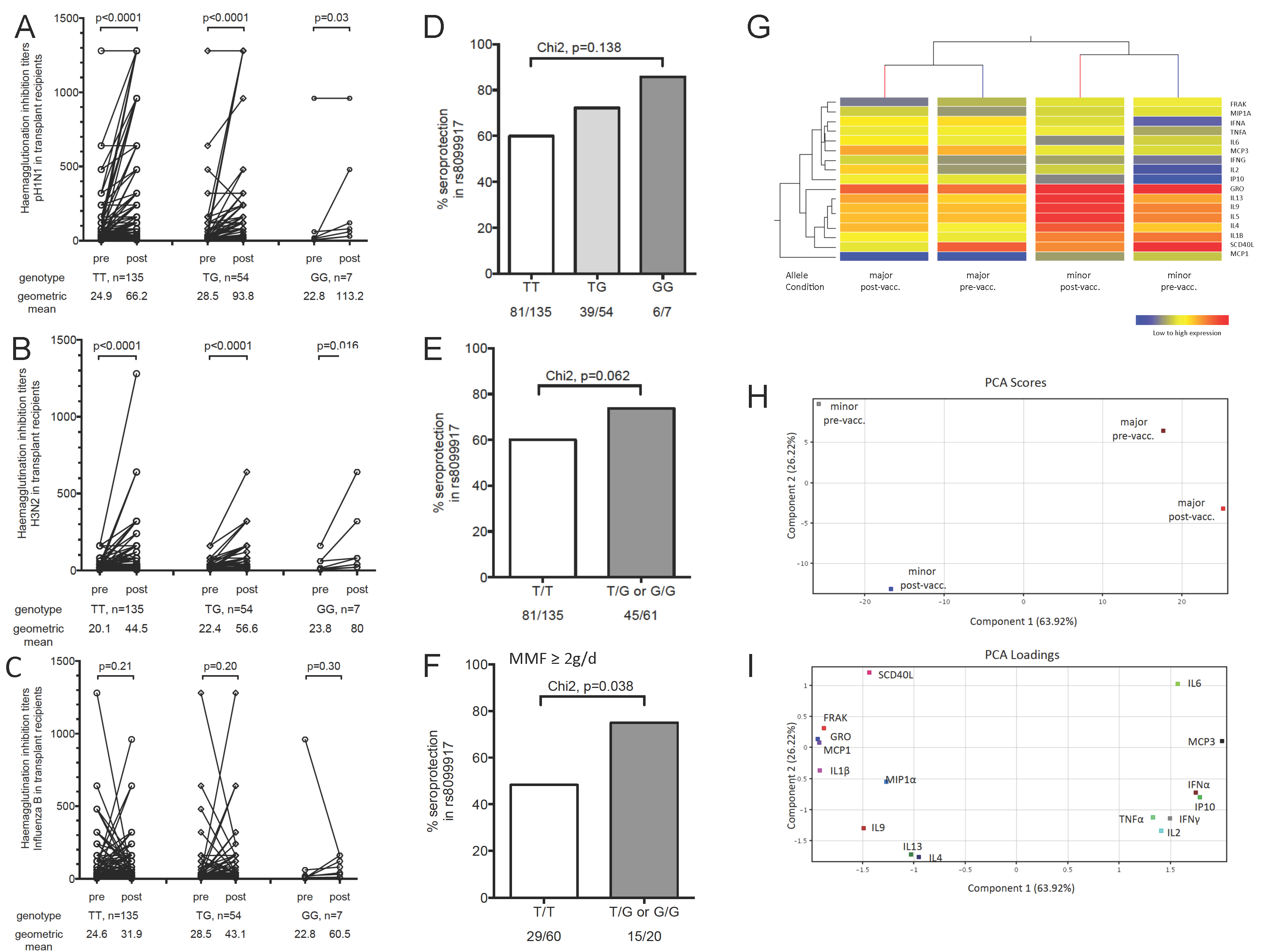

Supplement: S1 Figure — Analysis of H1N1-stimulated antibody and cytokine release in transplant recipients. (A–C) Pre- to post-vaccine haemagglutination inhibition (HAI) titer according to genotype. The HAI titers are shown for major allele carriers (TT, left) and minor allele carriers (no TT, right) for pH1N1 (A), H3N2 (B), and Influenza B (C). A total of 196 transplant recipients is shown. (D–F) Seroprotection to at least two vaccine antigens. (D) Percent seroprotection to at least two influenza strain antigens in T/T (major) versus T/G or G/G (minor) IL-28B SNP in transplant recipients (rs8099917). Chi2 test was used to determine significance. (E) Percent seroprotection to at least two influenza strain antigens in T/T vs. T/G vs. G/G IL-28B SNP in transplant recipients (rs8099917). (F) Percent seroprotection to at least two influenza strain antigens in T/T (major) versus T/G or G/G (minor) IL-28B SNP in transplant recipients (rs8099917) receiving 2 g or more mycophenolate mofetil (MMF) per day. (G–I) Supernatants of H1N1 stimulated PBMCs from transplant recipients (n = 47) was collected after 18 h. A luminex-based cytokine profile analysing 17 cytokines was performed. (G) Clustering analysis comparing IL-28B genotype pre- and post-vaccine (vacc.). (H) Principal component analysis showing PCA scores dependent on IL-28B genotype and pre- vs. post-vaccine state (2-dimensional). (I) Principal component analysis showing PCA loadings of cytokines dependent on IL-28B genotype and pre- vs. post-vaccine state (2-dimensional). (TIFF) [file ppat.1004556.s001.tiff]

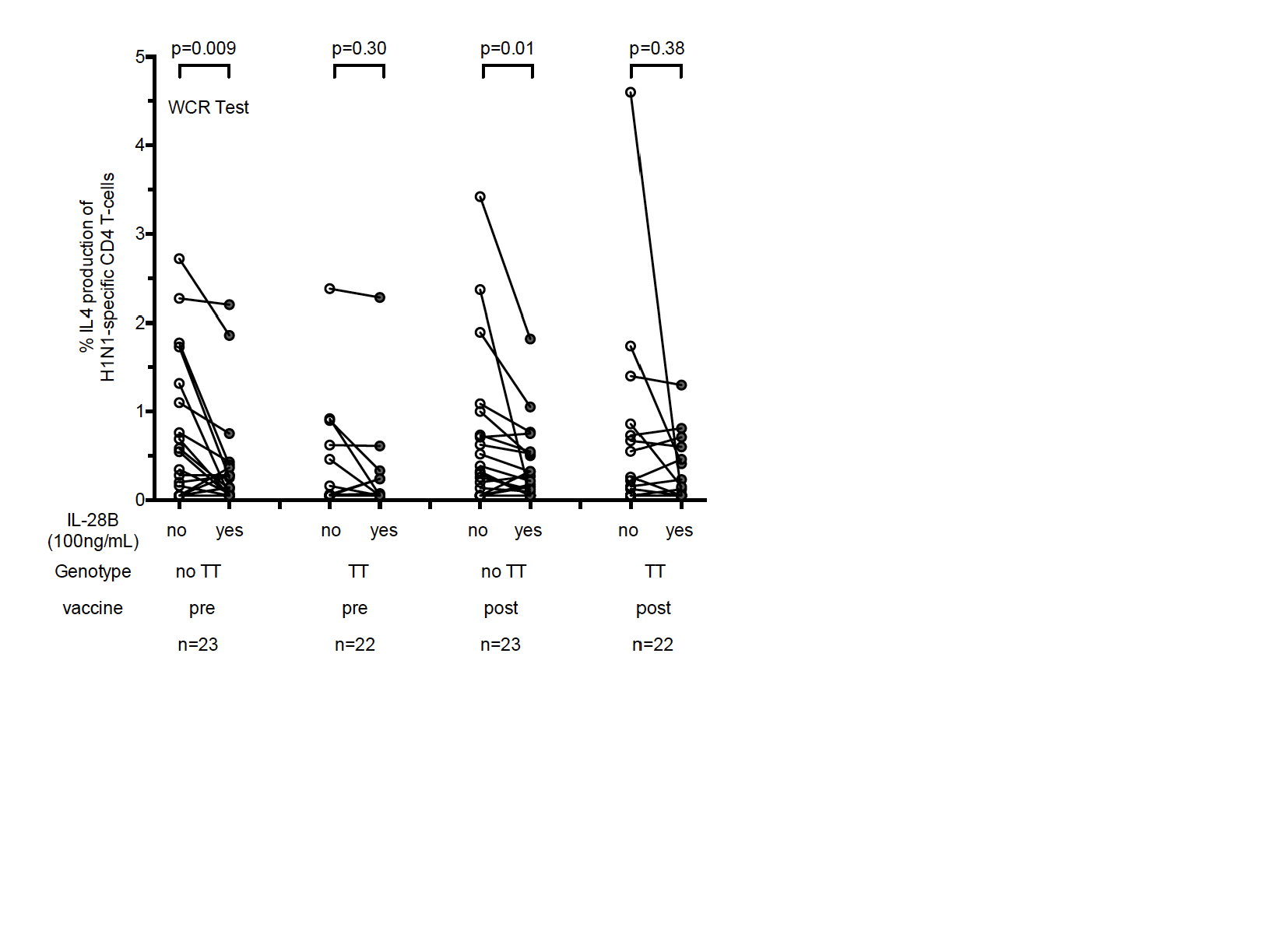

Supplement: S2 Figure — Frequency of IL-4-producing CD4 T-cells and impact of pre-treatment with IL-28B. The differential effect of IL-28B genotype background in PBMCs before and after vaccination is shown. In particular, PBMCs with a minor allele background (no TT) show a strong sensitivity to IL-28B. Wilcoxon matched pairs rank test was used to test for significant difference. Data from 45 transplant recipients is shown. (TIFF) [file ppat.1004556.s002.tiff]

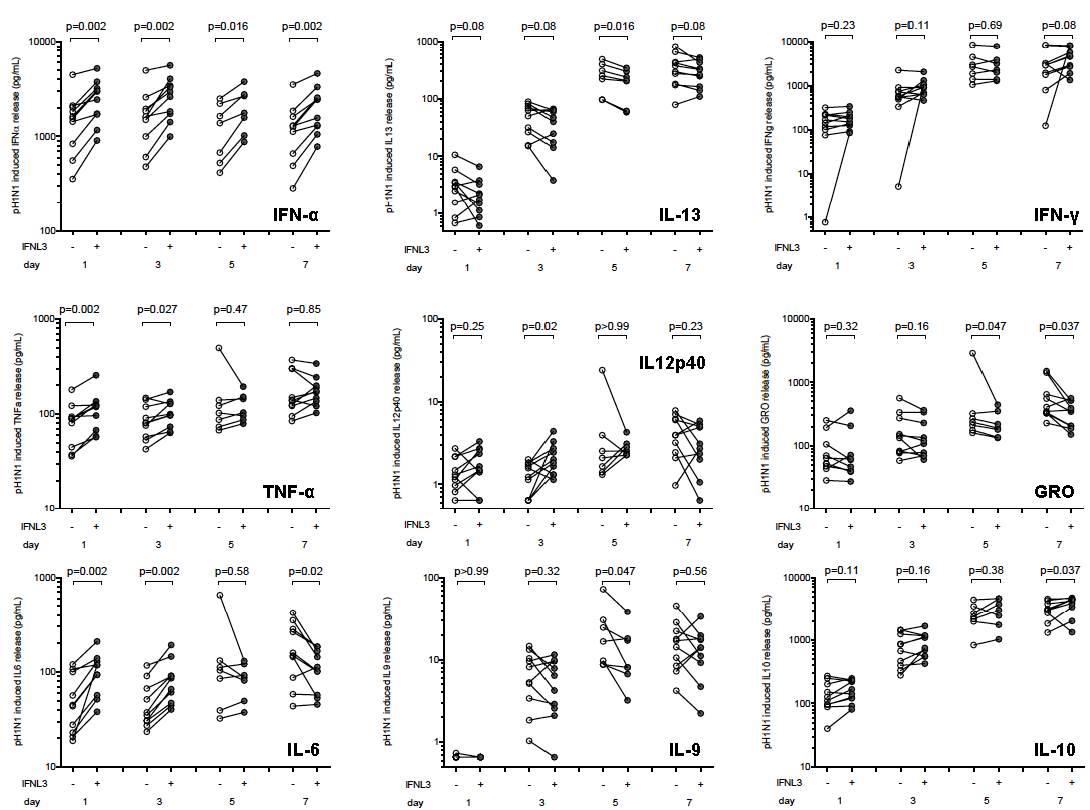

Supplement: S3 Figure — A cytokine profile with pH1N1 stimulation +/− IL-28B pre-treatment (100 ng/ml) was performed. The time-points were collected within the same experiments. For the first 3 HVs studied, d+5 sample was not collected. Overall, the time-points d+1, d+3, d+7 were available in total 10 HVs and for d+5 in 7 HVs. Wilcoxon matched pairs rank test was used to test for significant difference. (TIFF) [file ppat.1004556.s003.tiff]

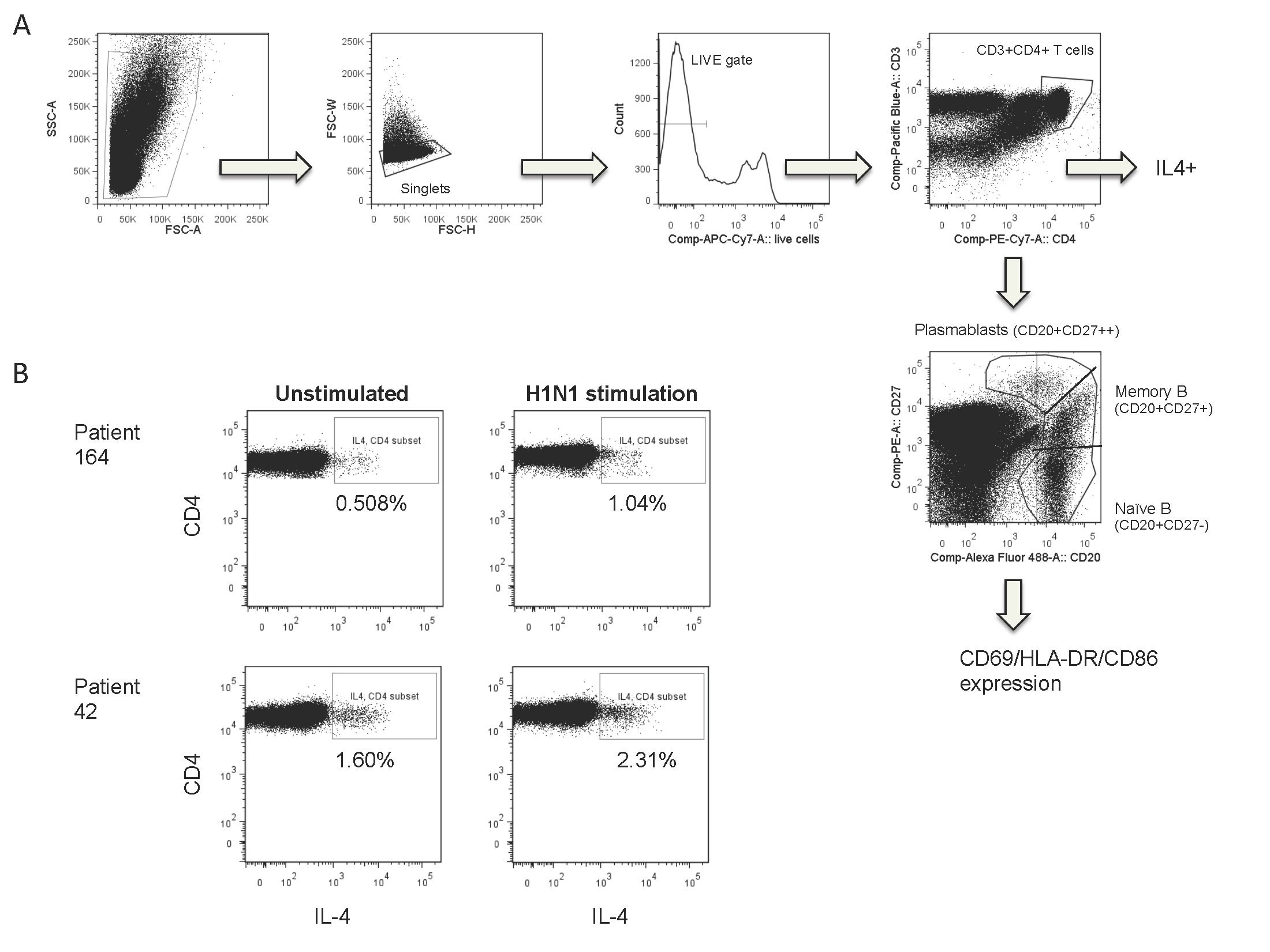

Supplement: S4 Figure — Gating strategy for flow cytometry analysis of H1N1-specific T-cell and B-cell responses. (A) After exclusion of singlets and dead cells, either T-cells (CD3+CD4+), or B-cells (CD20+CD27−, naïve B-cells; CD20+CD27+, memory B-cells; CD20+CD27++, plasma cells/plasmablasts) were gated. In T-cells, intracellular cytokine staining for IL-4+ was determined. Cytokine producing T-cell subsets were expressed as the % frequency of overall CD3+CD4+ T-cells. In B-cells, surface staining with CD86, HLA-DR and CD69 was determined. Mean fluorescence intensity was used to express the surface expression of the respective activation marker. Background expression (non-stimulated cells) was subtracted for all experiments. (B) Examples of IL-4 producing H1N1-stimulated and non-stimulated CD4+ T-cells are shown. For further analysis the non-stimulated background sample was subtracted. (TIFF) [file ppat.1004556.s004.tiff]

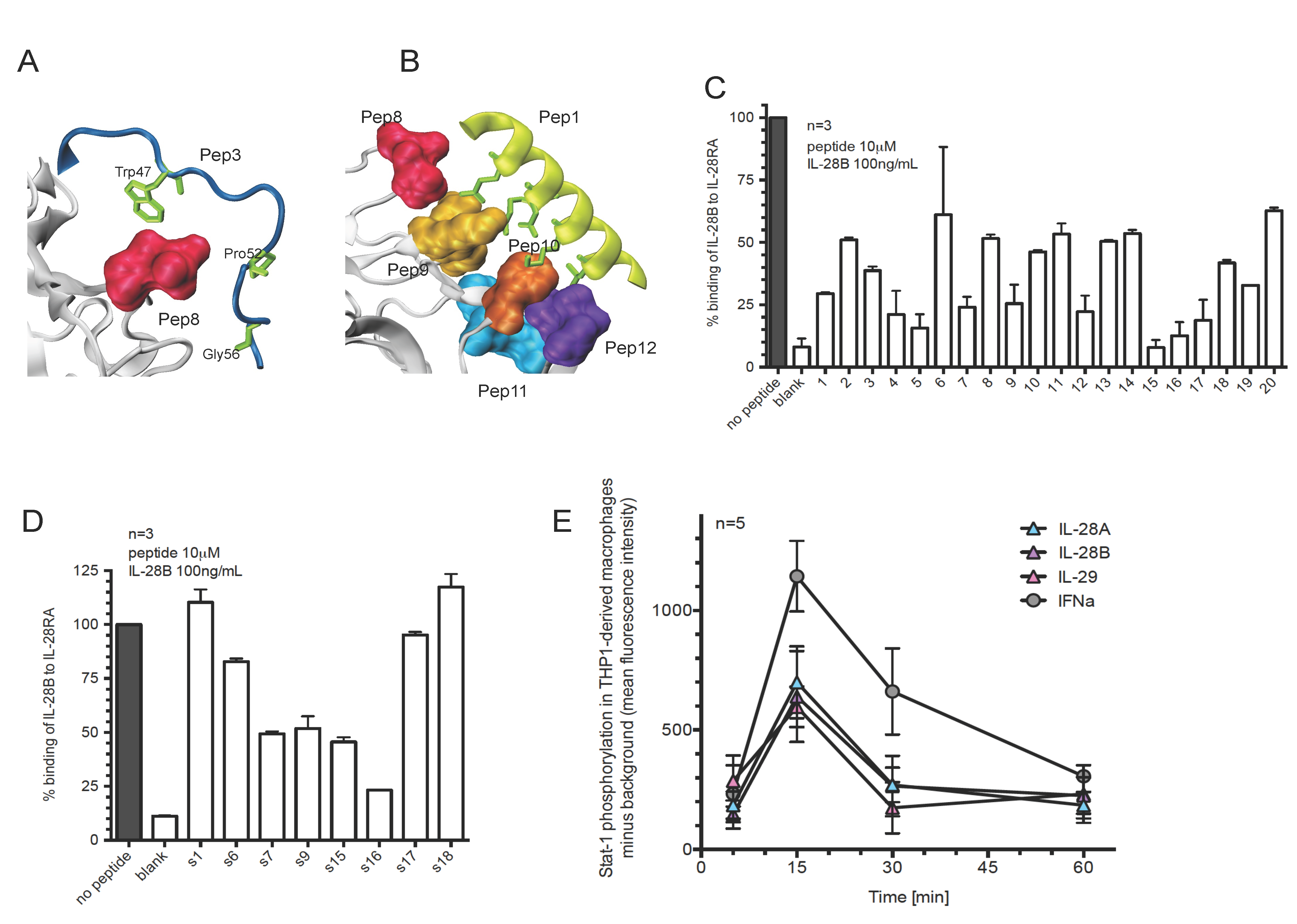

Supplement: S5 Figure — Design of antagonistic peptides to IL-28 receptor (IL28RA) and their binding affinity. (A) Exampled of detailed in silico interaction focusing on peptide 3 and IL28RA. (B) Exampled of detailed in silico interaction focusing on peptide 1 and IL28RA. (C) Inhibitory activity of peptides against IL-28B to IL28RA. ELISA was used to measure the binding of a fixed concentration of IL-28B (100 ng/mL) to IL28RA challenged by a fixed concentration of antagonistic peptides (10 µM). Bars indicate median values, whiskers inter-quartile ranges. The graph represents three independently repeated experiments. (D) Inhibitory activity of scrambled peptides against IL-28B to IL28RA. ELISA was used to measure the binding of a fixed concentration of IL-28B (100 ng/mL) to the IL28RA challenged by a fixed concentration of control peptides (10 µM; s, scramble). The median value representing three independently repeated experiments is shown. Whiskers indicate the interquartile range. (E) Time-course of STAT-1 phosphorylation in THP1-derived macrophages peak at 15 min. STAT-phosphorylation is expressed in mean fluorescent intensities of 5 independent experiments, mean and SEM is shown. (TIFF) [file ppat.1004556.s005.tiff]

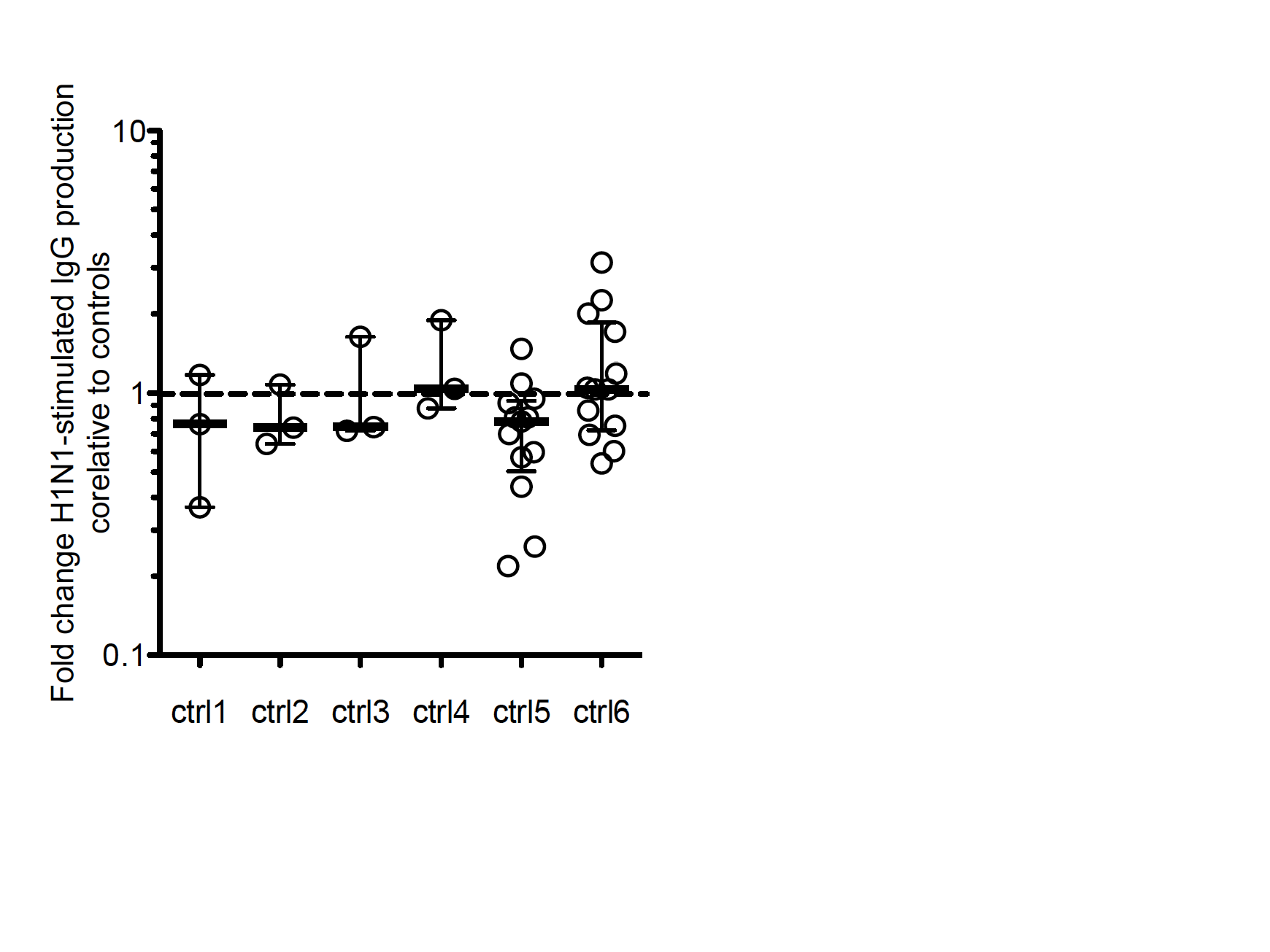

Supplement: S6 Figure — Effects of control peptides on H1N1 stimulated IgG production. Various control peptides were added twice to H1N1 stimulated PBMCs to maximize a potential unspecific stimulatory effect during the expansion phase. No unspecific effect could be observed. Control peptides are abbreviated as “ctrl”. Ctrl 1–3, “DV2”, “DV8”, “DV10” are peptides based on the NS5A of HCV, Ctrl 4, “DHBV” is a peptide based on the pre S region of duck hepatitis B virus encoded proteins. Ctrl 5, “Pen” is penetratin from drosophila, and Ctrl 6, “SV40” is based on the large T protein in simian virus 40. All control peptides are unrelated to IL-28A, IL-28B, IL-29 or IL28RA or IL10RB. (TIFF) [file ppat.1004556.s006.tiff]
