# Supplementary material for: IL-28B is a Key Regulator of B- and T-Cell Vaccine Responses against Influenza
Source: PLoS Pathog. 2014 Dec 11;10(12):e1004556. doi: 10.1371/journal.ppat.1004556 (PMC4263767; doi:10.1371/journal.ppat.1004556)
Supplement: S1 Table — Demographics of transplant cohort. (DOCX) [file ppat.1004556.s007.docx]

**Table S1. Demographics of transplant cohort.**

| **Characteristics** | **All (n=196)** | **IL-28B major-allele^e^**  **(TT; n=135)** | **IL-28B minor-allele^e^**  **(no TT; n=61)** | **p-values** |
| --- | --- | --- | --- | --- |
| Age; median (range) in years^a^ | 54.3 (19.7-76.9) | 54.3 (45.5-61.6) | 56.3 (45.9-67.1) | 0.279 |
| Gender; male/female | 143/53 | 97/38 | 46/15 | 0.604 |
| Time post transplant;  median (range) in years | 4.9 (0.2 – 32.4) | 4.54 (1.29-8.75) | 4.44 (1.64-10.02) | 0.572 |
| Type of vaccine (i.m./i.d.) | 95/101 | 66/69 | 29/32 | 0.861 |
| Pre-vaccine geometric mean titer,  % seroprotected |  |  |  |  |
| pH1N1 |  | 24.3, 44.4% | 27.8, 36.1% | 0.650 |
| H3N2 |  | 20.3, 43% | 22.5, 44.3% | 0.560 |
| Influenza B |  | 17.9, 34.8% | 20.8, 41.0% | 0.696 |
| Type of transplant |  |  |  |  |
| Lung | 64 (32.3%) | 44 (32.6%) | 20 (32.8%) | 0.391 |
| Kidney | 91 (41.0%) | 67 (49.6%) | 24 (39.3%) |  |
| Heart | 17 (7.9%) | 10 (7.4%) | 7 (11.5%) |  |
| Liver | 24 (11.4%) | 14 (10.4%) | 10 (16.4%) |  |
| Immunosuppression^c^ |  |  |  |  |
| Prednisone (%) | 143 (73%) | 101 (74.8%) | 42 (68.9%) | 0.384 |
| Tacrolimus (%) | 147 (75%) | 103 (76.3%) | 44 (72.1%) | 0.533 |
| MMF dose g/d, median |  | 2 (1.04-2) | 2 (1-2) | 0.646 |
| MMF^d^ ≥2g/d (%) | 80 (40.2%) | 60 (44.4%) | 20 (32.8%) | 0.124 |

^a^ Age at time of enrolment into study.

^b^ Type of vaccine: intra-muscular versus intra-dermal vaccine application.

^c^ Immunosuppressive treatment at time of vaccination.

^d^ MMF = mycophenolate mofetil.

^e^ Genotype for rs8099917, a total of 196 patients were genotyped.

All listed variables between TT and no TT did not show any significant difference.
