# Supplementary material for: IL-28B is a Key Regulator of B- and T-Cell Vaccine Responses against Influenza
Source: PLoS Pathog. 2014 Dec 11;10(12):e1004556. doi: 10.1371/journal.ppat.1004556 (PMC4263767; doi:10.1371/journal.ppat.1004556)
Supplement: S2 Table — Distribution of allele frequencies of IL-28B genotypes (rs8099917 and rs12979860) in transplant cohort. (DOCX) [file ppat.1004556.s008.docx]

**Table S2.** **Distribution of allele frequencies of IL-28B genotypes (rs8099917 and rs12979860) in transplant cohort.**

|  |  | **rs8099917^b^** |  |  |  |  |
| --- | --- | --- | --- | --- | --- | --- |
|  |  | **T/T** | **G/T** | **G/G** |  | **Total** |
| **rs12979860^a^** | **C/C** | 91 (46.7%) | 2 (1.0%) | 0 |  | 93 |
|  | **C/T** | 37 (19.0%) | 47 (24.1%) | 0 |  | 84 |
|  | **T/T** | 6 (3.1%) | 5 (2.6%) | 7 (3.6%) |  | 18 |
|  |  |  |  |  |  |  |
|  | **Total** | 134 | 54 | 7 |  | 195 |

^a^ rs12979860; C/C major-allele, C/T or T/T minor-allele genotype.

^b^ rs8099917; T/T major-allele, G/T or G/G minor-allele genotype.

For 195/196 patients both SNPs were available to be included into the cross-table analysis.
