# Supplementary material for: IL-28B is a Key Regulator of B- and T-Cell Vaccine Responses against Influenza
Source: PLoS Pathog. 2014 Dec 11;10(12):e1004556. doi: 10.1371/journal.ppat.1004556 (PMC4263767; doi:10.1371/journal.ppat.1004556)
Supplement: S3 Table — Influenza strain specific seroconversion rates in relation to IL-28B genotype. (DOCX) [file ppat.1004556.s009.docx]

**Table S3. Influenza strain specific seroconversion rates in relation to IL-28B genotype.**

| **IL-28B Genotype** | **Seroconversion H1N1** | | **Chi^2^**  **p-value** | **Seroconversion H3N2** | | **Chi^2^**  **p-value** | **Seroconversion**  **Influenza B** | | **Chi^2^**  **p-value** |
| --- | --- | --- | --- | --- | --- | --- | --- | --- | --- |
| rs8099917 | no | yes |  | No | yes |  | no | yes |  |
| T/T | 92 (68.2%) | 43 (31.9%) | 0.09 | 100 (74.1%) | 35 (25.9%) | 0.06 | 111 (82.2%) | 24 (17.8%) | 0.40 |
| T/G or G/G | 34 (55.7%) | 27 (44.3%) |  | 37 (60.7%) | 24 (39.3%) |  | 47 (77.1%) | 14 (23%) |  |
|  |  |  |  |  |  |  |  |  |  |
| rs12979860 | no | yes |  | no | yes |  | no | yes |  |
| C/C | 62 (66.0%) | 32 (34%) | 0.71 | 75 (79.8%) | 19 (20.2%) | 0.94 | 68 (72.3%) | 26 (27.7%) | 0.64 |
| C/T or T/T | 64 (63.4%) | 37 (36.6%) |  | 81 (80.2%) | 20 (19.8%) |  | 70 (69.3%) | 31 (30.7%) |  |
