# Supplementary material for: IL-28B is a Key Regulator of B- and T-Cell Vaccine Responses against Influenza
Source: PLoS Pathog. 2014 Dec 11;10(12):e1004556. doi: 10.1371/journal.ppat.1004556 (PMC4263767; doi:10.1371/journal.ppat.1004556)
Supplement: S4 Table — Seroconversion rates to at least one antigen of influenza vaccine in relation to IL-28B genotype. (DOCX) [file ppat.1004556.s010.docx]

**Table S4. Seroconversion rates to at least one antigen of influenza vaccine in relation to IL-28B genotype.**

|  | **Seroconversion to**  **at least one influenza vaccine antigen** | | | **Chi^2^,**  **p-value** |
| --- | --- | --- | --- | --- |
| **IL-28B SNP rs8099917** | no | yes | Total |  |
| **T/T (major)** | 76 (56.3%) | 59 (43.7%) | 135 | **0.028** |
| **T/G or G/G (minor)** | 24 (39.3%) | 37 (60.7%) | 61 |  |
|  | 100 | 96 | 196 |  |
|  |  |  |  |  |
| **IL-28B SNP rs12979860** | no | yes | Total |  |
| **C/C (major)** | 47 (50.0%) | 47 (50.0%) | 94 | 0.730 |
| **C/T or T/T (minor)** | 53 (52.5%) | 48 (47.5%) | 101 |  |
|  | 100 | 95 | 195^a^ |  |

^a^ For rs12979860 only 195 of 196 patients could be genotyped.
