# Supplementary material for: IL-28B is a Key Regulator of B- and T-Cell Vaccine Responses against Influenza
Source: PLoS Pathog. 2014 Dec 11;10(12):e1004556. doi: 10.1371/journal.ppat.1004556 (PMC4263767; doi:10.1371/journal.ppat.1004556)
Supplement: S5 Table — The effects of IL-28B genotype on production of cytokines by transplant recipient PBMCs in response to in vitro overnight influenza H1N1 stimulation. (DOCX) [file ppat.1004556.s011.docx]

**Table S5.** **The effects of IL-28B genotype on production of cytokines by transplant recipient PBMCs in response to *in vitro* overnight influenza H1N1 stimulation.**

| **Cytokine, pg/mL^a^** | **Minor SNP IL-28B (no TT)** | **Major SNP IL-28B (TT)** | **Ratio Major/minor** | **p-value^c^** |
| --- | --- | --- | --- | --- |
| Fractalkine | 3.2 (3.2-19.4)^b^ | 3.2 (3.2-60.8) | 1.0 | 0.61 |
| IFN-α | 210.7 (14.0-715.9) | 602.8 (295.6-879.3) | 2.9 | 0.15 |
| IFN-γ | 9.8 (0.6-46.2) | 37.5 (11.0-58.6) | 3.8 | 0.13 |
| GRO | 3.2 (3.2-3.2) | 3.2 (3.2-3.2) | 1.0 | 1.00 |
| MCP-3 | 336.9 (75.1-702.6) | 668.9 (551.9-2524.7) | 2.0 | 0.09 |
| IL-13 | 0.6 (0.6-0.7) | 0.6 (0.6-1.0) | 1.0 | 0.92 |
| sCD40-L^d^ | 0.6 (0.6-0.6) | 0.6 (0.6-9.6) | 1.0 | 0.66 |
| IL-9 | 0.6 (0.6-0.6) | 0.6 (0.6-0.6) | 1.0 | 1.00 |
| IL-1β | 0.6 (0.6-1.2) | 1.2 (0.6-3.8) | 2.0 | 0.09 |
| IL-2 | 32.0 (6.8-106.3) | 81.5 (26.9-198.6) | 2.5 | 0.09 |
| IL-4 | 0.6 (0.6-0.6) | 0.6 (0.6-1.0) | 1.0 | 0.90 |
| IL-5 | 0.6 (0.6-0.6) | 0.6 (0.6-0.6) | 1.0 | 1.00 |
| IL-6 | 8.7 (0.5-51.0) | 77.0 (14.7-144.1) | **8.9** | **0.02** |
| IP-10 | 6481.8 (152.8-8488.2) | 8847.0 (5222.3-9512.0) | **1.4** | **0.03** |
| MCP-1 | 0.5 (0.5-1424.6) | 0.5 (0.5-1443.0) | 1.0 | 0.62 |
| MIP-1α | 108.1 (3.2-381.8) | 204.6 (98.2-319.2) | 1.9 | 0.53 |
| TNF-α | 2.3 (0.7-37.4) | 20.6 (5.4-30.4) | 9.0 | 0.47 |

^a^ Cytokine secretion (as measured by multiplex cytokine analysis) from PBMCs from transplant recipients (n=47) stimulated with inactivated Influenza A H1N1 (0.3µg/mL hemagglutinin) overnight, independent of seroconversion.

^b^ Median values and inter-quartile ranges (IQR) are shown

^c^ Wilcoxon matched-pairs signed rank test

^d^ Soluble CD40 ligand
