# Supplementary material for: IL-28B is a Key Regulator of B- and T-Cell Vaccine Responses against Influenza
Source: PLoS Pathog. 2014 Dec 11;10(12):e1004556. doi: 10.1371/journal.ppat.1004556 (PMC4263767; doi:10.1371/journal.ppat.1004556)
Supplement: S6 Table — The effects of IL-28B treatment on production of cytokines by transplant recipient PBMCs in response to in vitro overnight influenza H1N1 stimulation. (DOCX) [file ppat.1004556.s012.docx]

**Table S6. The effects of IL-28B treatment on production of cytokines by transplant recipient PBMCs in response to *in vitro* overnight influenza H1N1 stimulation.**

| **Cytokine, pg/mL^a^** | **No IL-28B, Median pg/ml (IQR)**^b^ | **IL-28B (100ng/mL), Median pg/ml (IQR)** | **Ratio IL-28B/no IL-28B** | **p-value^c^** |
| --- | --- | --- | --- | --- |
| Fractalkine | 3.2 (3.2-45.2) | 16.1 (3.2-60.2) | 5.0 | 0.39 |
| IFN-α | 450.8 (98.4-764.8) | 1290.8 (502.4-1969.9) | **2.9** | **0.001** |
| IFN-γ | 23.4 (0.6-58.1) | 48.0 (13.9-133.3) | **2.1** | **0.04** |
| GRO | 3.2 (3.2-3.2) | 3.2 (3.2-3.2) | 1.0 | 1.00 |
| MCP-3 | 632.7 (226.9-1865.8) | 760.9 (398.3-2500.5) | 1.2 | 0.33 |
| IL-13 | 0.6 (0.6-0.9) | 0.6 (0.6-0.9) | 1.0 | 1.00 |
| sCD40-L^d^ | 0.6 (0.6-5.2) | 0.6 (0.6-77.7) | **1.0** | **0.04** |
| IL-9 | 0.6 (0.6-0.6) | 0.6 (0.6-0.6) | 1.0 | 1.00 |
| IL-1β | 0.6 (0.6-2.4) | 0.6 (0.6-4.0) | 1.0 | 0.44 |
| IL-2 | 56.2 (7.3-163.5) | 61.5 (6.0-168.9) | 1.1 | 0.86 |
| IL-4 | 0.6 (0.6-0.6) | 0.6 (0.6-1.5) | 1.0 | 0.92 |
| IL-5 | 0.6 (0.6-0.6) | 0.6 (0.6-0.6) | 1.0 | 1.00 |
| IL-6 | 42.8 (0.5-116.0) | 104.0 (15.0-156.6) | **2.4** | **0.05** |
| IP-10 | 8122.4 (2441.2-9176.5) | 8336.4 (7362.5-9289.1) | 1.0 | 0.14 |
| MCP-1 | 0.5 (0.5-1454.1) | 0.5 (0.5-827.4) | 1.0 | 0.61 |
| MIP-1α | 173.9 (3.2-377.2) | 321.4 (173.7-485.3) | **1.8** | **0.04** |
| TNF-α | 12.5 (0.7-33.1) | 23.2 (0.7-44.8) | 1.9 | 0.39 |

^a^ Cytokine secretion (as measured by multiplex cytokine analysis) from PBMCs from transplant recipients (n=47) stimulated with inactivated Influenza A H1N1 (0.3µg/mL hemagglutinin) overnight, independent of seroconversion.

^b^ Median values and inter-quartile ranges (IQR) are shown

^c^ Wilcoxon matched-pairs signed rank test

^d^ Soluble CD40 ligand
