# Supplementary material for: IL-28B is a Key Regulator of B- and T-Cell Vaccine Responses against Influenza
Source: PLoS Pathog. 2014 Dec 11;10(12):e1004556. doi: 10.1371/journal.ppat.1004556 (PMC4263767; doi:10.1371/journal.ppat.1004556)
Supplement: S7 Table — The effects of IL-28B addition on production of cytokines by healthy volunteer PBMCs in response to in vitro influenza H1N1 stimulation. (DOCX) [file ppat.1004556.s013.docx]

**Table S7. The effects of IL-28B addition on production of cytokines by healthy volunteer PBMCs in response to *in vitro* influenza H1N1 stimulation**

| **Cytokine (pg/mL) ^a^** | **No IL-28B, Median pg/ml (IQR)**^b^ | **With IL-28B, Median pg/ml (IQR)** | **Ratio IL28/no IL28** | **p-value^c^** |
| --- | --- | --- | --- | --- |
| EGF | 3.7 (3.7-3.7) | 3.7 (3.7-3.7) | 1.0 | 1.00 |
| Eotaxin | 6.5 (3.2-8.9) | 3.2 (3.2-6.9) | 0.5 | 0.47 |
| FGF2 | 17.0 (6.1-20.5) | 13.1 (3.8-19.2) | 0.8 | 0.31 |
| FLT3 | 7.9 (3.4-10.4) | 4.0 (3.2-9.4) | 0.5 | 0.11 |
| Fractalkine | **15.9** (5.6-25.7) | **4.2** (0.6-10.7) | **0.3** | **0.008** |
| G-CSF | 3.2 (3.2-3.2) | 3.2 (3.2-4.2) | 1.0 | 0.75 |
| GM-CSF | 486.9 (100.2-622.8) | 358.9 (3.2-480.7) | 0.7 | 0.38 |
| GRO | **146.1** (92.1-204.5) | **3.6** (3.6-3.6) | **0.0** | **0.008** |
| IFN-α2 | **1344.0** (245.4-2698.0) | **2262.0** (808.2-3663.0) | **1.7** | **0.004** |
| IFN-γ | 6892.0 (5076.0-7464.0) | 6028.0 (3513.0-9802.0) | 0.9 | 0.43 |
| IL-1α | 14.1 (7.3-17.4) | 15.1 (7.8-18.6) | 1.1 | 0.82 |
| IL-1β | **9.0** (4.7-11.8) | **16.8** (6.8-29.2) | **1.9** | **0.04** |
| IL-1ra | 334.3 (106.8-831.8) | 376.6 (3.2-838.8) | 1.1 | 0.84 |
| IL-2 | 8.1 (0.6-52.1) | 16.5 (0.6-60.4) | 2.0 | 0.84 |
| IL-3 | 3.0 (0.8-5.8) | 0.8 (0.6-3.5) | 0.3 | 0.16 |
| IL-4 | **32.9** (16.6-41.2) | **16.2** (6.1-33.1) | **0.5** | **0.03** |
| IL-5 | **21.6** (1.3-34.1) | **0.6** (0.6-8.3) | **0.0** | **0.02** |
| IL-6 | **170.5** (60.7-294.4) | **215.5** (69.4-864.5) | **1.3** | **0.03** |
| IL-7 | 3.2 (3.2-3.7) | 3.2 (3.2-3.2) | 1.0 | 0.75 |
| IL-8 | 1468.0 (0.5-2089.0) | 1308.0 (789.8-1567.0) | 0.9 | 0.95 |
| IL-9 | **8.1** (4.0-16.7) | **1.3** (0.6-6.7) | **0.2** | **0.008** |
| IL-10 | 2642.0 (2563.0-3352.0) | 2597.0 (2063.0-4353.0) | 1.0 | 0.82 |
| IL-12p40 | 4.3 (3.2-10.6) | 3.2 (3.2-6.1) | 0.7 | 0.44 |
| IL-12p70 | 2.4 (0.9-2.9) | 1.6 (1.0-2.3) | 0.7 | 0.64 |
| IL-13 | **174.4** (61.7-226.0) | **96.8** (0.6-126.7) | **0.6** | **0.02** |
| IL-15 | 2.9 (1.9-3.4) | 2.7 (1.2-3.0) | 0.9 | 0.17 |
| IL-17 | 3.5 (0.6-18.1) | 0.6 (0.6-30.7) | 0.2 | 0.69 |
| IP-10 | 697.4 (367.6-5086.0) | 219.8 (3.2-2804.0) | 0.3 | 0.08 |
| MCP-1 | 153.2 (0.6-174.5) | 53.8 (7.9-145.3) | 0.4 | 0.55 |
| MCP-3 | 8924.0 (8147.0-12731.0) | 9988.0 (8889.0-12741.0) | 1.1 | 0.25 |
| MDC | 16.0 (16.0-16.0) | 16.0 (16.0-27.2) | 1.0 | 1.00 |
| MIP-1α | 5020.0 (690.5-6797.0) | 5759.0 (2283.0-6915.0) | 1.1 | 0.74 |
| MIP-1β | 3096.0 (1715.0-6326.0) | 3575.0 (1795.0-5820.0) | 1.2 | 0.46 |
| PDGF AA | 0.6 (0.6 – 3.2) | 0.6 (0.6 – 0.6) | 1.0 | 0.25 |
| PDGF AB/BB | 6.9 (1.0-13.6) | 8.9 (1.3-18.2) | 1.3 | 0.95 |
| RANTES | 2992.0 (1318.0-4819.0) | 3640.0 (1941.0-6677.0) | 1.2 | 0.74 |
| sCD40L^d^ | **593.3** (279.7-818.7) | **253.0** (140.1-456.3) | **0.4** | **0.01** |
| sIL2Ra^d^ | **327.3** (187.0-353.5) | **192.2** (140.8-236.1) | **0.6** | **0.004** |
| TGF-α | **9.3** (4.6-17.1) | **11.8** (9.7-17.0) | **1.3** | **0.03** |
| TNF-α | 305.6 (0.6-781.7) | 297.0 (3.1-449.6) | 1.0 | 0.55 |
| TNF-β | 19.7 (0.7-36.3) | 12.0 (1.0-27.4) | 0.6 | 0.16 |
| VEGF | 29.0 (0.6-50.7) | 34.6 (15.6-46.3) | 1.2 | 0.82 |

^a^ Cytokine secretion (as measured by multiplex cytokine analysis) from PBMCs from healthy volunteers (n=9) stimulated with inactivated Influenza A H1N1 (0.3µg/mL hemagglutinin) for 5 days.

^b^ IQR= inter-quartile range

^c^ Wilcoxon matched-pairs signed rank test

^d^ soluble CD40 ligand or Interleukin 2 receptor
