# Supplementary material for: IL-28B is a Key Regulator of B- and T-Cell Vaccine Responses against Influenza
Source: PLoS Pathog. 2014 Dec 11;10(12):e1004556. doi: 10.1371/journal.ppat.1004556 (PMC4263767; doi:10.1371/journal.ppat.1004556)
Supplement: S8 Table — Amino acid sequences of Interferon-λ ligand or receptor-based inhibitory peptides. (DOCX) [file ppat.1004556.s014.docx]

**Table S8**. **Amino acid sequences of Interferon-λ ligand or receptor-based inhibitory peptides.**

| **Peptide derived from the ligand binding domains** | | | |
| --- | --- | --- | --- |
|  | Name | Sequence | Length |
| 1 | IL29-1 | ELASFKKARDALEESLKL | 18 |
| 2 | IL28-1 | ELQAFKRAKDALEESLLL | 18 |
| 3 | IL29-2 | LKNWSCSSPVFPGN | 14 |
| 4 | IL28A-2 | LKDCRCHSRLFPRT | 14 |
| 5 | IL28B-2 | LKDCKCRSRLFPRT | 14 |
| 6 | IL29-3 | ASVTFNLFRLLTRDLKY | 17 |
| 7 | IL28-3 | ASVTFNLFRLLTRDLNC | 17 |
| **Peptide derived from the receptor binding domains** | | | |
| 8 | IL28R-1 | VAYQSSPTRRRWREV | 15 |
| 9 | IL28R-2 | MMCLKKQDLYNKFKG | 15 |
| 10 | IL28R-3 | SEYLDYLFEVEPAPP | 15 |
| 11 | IL28R-4 | NATYQLPPCMPPLDLKY | 17 |
| 12 | IL28R-5 | ARTIYTFSVPKYSKF | 15 |
| **Peptides which interact with both ligand and receptor** | | | |
| 13 | IL28BT-1 | PQELQAFKRAKDALEESL | 18 |
| 14 | IL29T-1 | PQELASFKKARDALEESL | 18 |
| 15 | IL28BT-2 | LLKDCKCRSRLFPRTWDLRQ | 20 |
| 16 | IL28AT-2 | LLKDCRCHSRLFPRTWDLRQ | 20 |
| 17 | IL29T-2 | KLKNWSCSSPVFPGNWDLRL | 20 |
| 18 | IL28BT-3 | EATADTDPALGDVLDQPL | 18 |
| 19 | IL28AT-3 | EATADTDPALVDVLDQPL | 18 |
| 20 | IL29T-3 | EAAAGPALEDVLDQPL | 16 |
|  |  |  |  |
