# Supplementary material for: IL-28B is a Key Regulator of B- and T-Cell Vaccine Responses against Influenza
Source: PLoS Pathog. 2014 Dec 11;10(12):e1004556. doi: 10.1371/journal.ppat.1004556 (PMC4263767; doi:10.1371/journal.ppat.1004556)
Supplement: S9 Table — Primer and probe sequences for IL-28B SNP genotyping. (DOCX) [file ppat.1004556.s015.docx]

**Table S9**. **Primer and probe sequences for IL-28B SNP genotyping.**

| **Oligonucleotide** | **Sequence** |
| --- | --- |
| **rs12979860 SNP Taqman assay Life Technologies assay ID: AHS0QIE** | |
| rs12979860 SNP assay left hand primer | 5’-GCC TGT CGT GTA CTG AAC CA-3’ |
| rs12979860 SNP assay right hand primer | 5’-GCG CGG AGT GCA ATT CAA C-3’ |
| C allele probe | 5’-CTT CCG C**G**C TTG GT-3’ |
| T allele probe | 5’-CTT CCG C**A**C TTG GTC-3’ |
| **rs8099917 SNP Taqman assay Life Technologies assay ID: AH1RU99** | |
| rs8099917 SNP assay left hand primer | 5’-TCA CTG TTC CTC CTT TTG TTT TCCT-3’ |
| rs8099917 SNP assay right hand primer | 5’-CCA GCT ACC AAA CTG TAT ACA GCAT-3’ |
| G allele probe | 5’-TGT GAG CAA T**G**T CAC CC-3’ |
| T allele probe | 5’-TGT GAG CAA T**T**T CAC CC-3’ |
|  |  |
| **Positive control oligonucleotides** | |
| 49bp rs129709860 +’ve control C allele sense | 5’-GAA CCA GGG AGC TCC CCG AAG GCG **C**GA ACC AGG GTT GAA TTG CAC TCC G -3' |
| 49bp rs129709860 +’ve control C allele αsense | 5’-CGG AGT GCA ATT CAA CCC TGG TTC **G**CG CCT TCG GGG AGC TCC CTG GTT C -3' |
| 50bp rs129709860 +’ve control T allele sense | 5’-CTG AAC CAG GGA GCT CCC CGA AGG CG**T** GAA CCA GGG TTG AAT TGC ACT CC -3' |
| 50bp rs129709860 +’ve control T allele αsense | 5’-GGA GTG CAA TTC AAC CCT GGT TC**A** CGC CTT CGG GGA GCT CCC TGG TTC AG -3' |
| 50bp rs8099917 +’ve control G allele sense | 5’-CTT TTG TTT TCC TTT CTG TGA GCA AT**G** TCA CCC AAA TTG GAA CCA TGC TG-3’ |
| 50bp rs8099917 +’ve control G allele αsense | 5’-CAG CAT GGT TCC AAT TTG GGT GA**C** ATT GCT CAC AGA AAG GAA AAC AAA AG-3’ |
| 50bp rs8099917 +’ve control T allele sense | 5’-TTT TGT TTT CCT TTC TGT GAG CAA T**T**T CAC CCA AAT TGG AAC CAT GCT GT-3’ |
| 50bp rs8099917 +’ve control T allele αsense | 5’-ACA GCA TGG TTC CAA TTT GGG TGA **A**AT TGC TCA CAG AAA GGA AAA CAA AA-3’ |
